# LcProt: Proteomics‐based identification of plasma biomarkers for lung cancer multievent, a multicentre study

**DOI:** 10.1002/ctm2.70160

**Published:** 2025-01-09

**Authors:** Hengrui Liang, Runchen Wang, Ran Cheng, Zhiming Ye, Na Zhao, Xiaohong Zhao, Ying Huang, Zhanpeng Jiang, Wangzhong Li, Jianqi Zheng, Hongsheng Deng, Yu Jiang, Yuechun Lin, Yun Yan, Lei Song, Jie Li, Xin Xu, Wenhua Liang, Jun Liu, Jianxing He

**Affiliations:** ^1^ Department of Thoracic Surgery and Oncology the First Affiliated Hospital of Guangzhou Medical University State Key Laboratory of Respiratory Disease & National Clinical Research Center for Respiratory Disease Guangzhou China; ^2^ Department of Proteomics Tianjin Key Laboratory of Clinical Multi‐Omics Tianjin China

**Keywords:** lung cancer, multitask, plasma proteomics, zeolite NaY

## Abstract

**Background:**

Plasma protein has gained prominence in the non‐invasive predicting of lung cancer. We utilised Zeolite Zotero NaY‐based plasma proteomics to investigate its potential for multiple event predicting, including lung cancer diagnosis (task #1), lymph node metastasis detection (task #2) and tumour‒node‒metastasis (TNM) staging (task #3).

**Methods:**

A total of 4703 plasma proteins were quantified from 241 participants based on a prospective cohort of 2757 participants. An additional 46 participants from external prospective cohort of 735 participants were used for validation. Feature selection was performed using differential expressed protein analysis, area under curve (AUC) evaluation and least absolute shrinkage and selection operator (LASSO) regression. Random forest was used for multitask model construction based on the key proteins. Feature importance was interpreted using Shapley additive explanations (SHAP) algorithm.

**Results:**

For task #1, 10 proteins panel showed an AUC of .87 (.77‒.97) in the external validation. After integrating clinical factors, a significant increase diagnostic accuracy was observed with AUC of .91 (.85‒.98). For task #2, nine proteins panel achieved an AUC of .88 (.80‒.96), integration model showed an increase diagnostic accuracy with AUC of .90 (.85‒.97). For task #3, 10 proteins panel showed an AUC of .88 (.74‒.96) for stage I, .92 (.84‒.97) for stage II, .88 (.76‒.96) for stage III and .99 (.98‒.99) for stage IV in the integration model.

**Conclusions:**

This study comprehensively profiled the NaY‐based plasma proteome biomarker, laying the foundation for a high‐performance blood test for predicting multiple events in lung cancer.

**Key points:**

- Our study developed an innovative nanomaterial, Zeolite NaY, which addressed the masking effect and improved the depth of the proteome.
- The performance of NaY‐based plasma proteomics as a preclinical diagnostic tool was validated through both internal and external cohort.
- Furthermore, we explored the different patterns of plasma protein changes during the progression of lung cancer and used the explanations method to elucidate the roles of proteins in the multitask predictive model.

## Graphical abstract

•NaY‐based proteomics enables cost‐effective and efficient plasma proteome screening.

•Prospective cohorts uncover plasma protein dynamics across benign and malignant nodules and different cancer stages.

•Machine learning models achieve high‐accuracy lung cancer diagnosis from plasma profiles.

•A minimal biomarker panel identified via NaY‐based proteomics enables low‐cost, multitasking capabilities for diagnosis and staging.

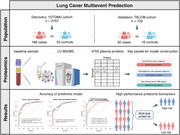

## 1 INTRODUCTION

Lung cancer is the leading cause of cancer‐related deaths worldwide.^1^ Dynamic monitoring the present and development of lung cancer is of great importance. Non‐invasive biomarker such as circulating tumour DNA, DNA methylation, microRNA and autoantibody have proven essential in identifying multiple events in lung cancer, including screening, staging and lymph node metastasis detection.^2^, ^3^, ^4^, ^5^, ^6^, ^7^, ^8^, ^9^, ^10^

Recently, proteomics has gained prominence in non‐invasive monitoring methods due to its advantageous features, including higher accuracy, stability and functionality, and cost‐effectiveness.^11^ However, current study using conventional mass spectrometry (MS) method for liquid biopsy has certain drawbacks. First, the depth of conventional MS technique is limited, resulting in a restricted number of proteins available for subsequent analysis.^12^ Second, high‐abundance proteins masking effect can hinder the identification of low‐abundance proteins, which may be equally important.^13^, ^14^, ^15^ Third, the focused on a single endpoint limits its clinical application.

To overcome these shortcomings, our research team previously introduced a novel nano adsorbent, zeolite NaY, which possesses combined physicochemical to capture a broader range of proteins. Compared with conventional MS method, this innovation provided more in‐depth MS results and effectively addressed the masking effect during plasma proteome analysis.^16^

Here, we present a prospective, multicentre, multitask predictive model, named LcProt, to validate the potential roles of zeolite NaY‐based MS techniques in various lung cancer time point events. The multitask included lung cancer diagnosis (task #1), lymph node metastasis detection (task #2) and tumour‒node‒metastasis (TNM) staging (task #3). We synthesised zeolite NaY and developed a rapid, and reproducible method based on the nanomaterial zeolite NaY.

## 2 METHODS

### 2.1 Patient selection

Guangzhou Medical University (GMU) cohort is a prospective, multicentre cohort, which includes 2757 participants with various lung disease. These participants consist of surgically treatment non‐small cell lung cancer (NSCLC) patients, NSCLC and small cell lung cancer patients receiving comprehensive treatment, as well as patients with benign lung diseases undergoing routine physical examinations. Peripheral blood samples before and after treatment, tumour tissue, pair para‐cancerous tissue and biopsy tissue samples were planned to collect based on the treatment protocols. A detailed description of the GMU cohort was provided in Supporting Information S1.

We prospectively and consecutively collected data from 127 participants from the First Affiliated Hospital of Guangzhou Medical University (1STGMU) cohort, one of the sub‐cohorts in the GMU cohort, including 106 lung cancer patients and 21 benign lung disease patients. A total of 101 participants were selected from another participating centre, the National Center for Respiratory Medicine (NCRM) cohort, comprising 75 lung cancer patients and 26 patients with benign lung disease. Tianjin Key Laboratory of Clinical Multi‐omics (TKLCM) another prospective cohort contains 735 participants, we selected 30 lung cancer patients and 16 patients with benign lung disease for external validation.

Inclusion criteria were: (1) 18 years of age or older, (2) histopathologically diagnosed lung cancer or benign lung disease, (3) underwent radical surgical resections with systemic intrathoracic lymph node dissection, and (IV) availability of clinicopathological data.

Exclusion criteria were: (1) patients with severe systemic disease, (2) history of previous malignancy within the past 5 years, and (3) received previous systemic antitumour therapies.

Lung cancer and benign lung disease diagnosis was confirmed through histopathologically examination. Baseline data, including gender, age, body mass index (BMI), underlying diseases, smoking status, TNM stage, tumour location, tumour size, genetic mutation and levels of carbohydrate antigen (CA)‐125, CA‐153 and carcinoembryonic antigen (CEA) were collected for all patients.

### 2.2 Sample preparation and kit preparation

Collected blood samples were used to prepare calibration and quality control (QC) samples. Two specific QCs were implemented throughout the sample preparation and MS detection processes. The first, sample preparation QC, involved preparing pooled plasma samples alongside test samples withing each batch. Repeated testing of pooled samples was used to assess consistency in samples preparation across batches. The second, QC‐MS, involved preparing long‐lasting peptide samples. One QC‐MS sample was included after every 20 cohort samples to monitor the stability of the MS instrument.

For each patient, 5 mL of peripheral blood was obtained through venipuncture using a vacuum blood collection tube containing ethylenediaminetetraacetic acid anticoagulant. All blood samples were transported to the clinical laboratory within 4 h and centrifuged at 1600 *g* for 15 min with 4°C using a centrifuge. Following centrifugation, upper plasma layers were collected and stored in a freezer at ‒80°C until further analysis. Extensive information about standard operating procedures can be found in the Supporting Information S2.

The nanomaterial NaY used in this study was derived from the nano‐low abundant plasma enrichment/MS TM through gel formulation and calcination by the TKLCM (Figure S1). Detailed of the synthesis process for these nanomaterials were detailed in our previous work.^16^ Detailed kit preparation method was described in the Supporting Information S3.

### 2.3 Liquid chromatography‒mass spectrometry/mass spectrometry

Equal amounts of indexed retention time were added to the peptide samples, which were then analysed using the Thermo Scientific UltiMate 3000 UHPLC system coupled with an Orbitrap Q Exactive HF mass spectrometer. The analysis utilised a gradient elution as follows: 0‒5 min, 3%‒6% B; 6‒44 min, 6%‒90% B; 45‒54 min, 90% B; 55‒60 min. The flow rate was maintained at 600 nL/min, and separation was achieved on a 150 µm ID × 30 cm column (C18, 1.9 µm, 120 Å, Dr. Maisch GmbH). The spray voltage was set at 2000 V in positive ion mode, with the ion transfer tube temperature at 320°C.

The mass spectrometer operated in data‐independent acquisition (DIA) mode, with an m/z scan range of 350‒1500 and a resolution of 60 000. The automatic gain control target was set at 1 × 10^6^ with an automatic maximum injection time setting. High‐energy collisional dissociation fragmentation was performed at a resolution of 30 000, with a normalised collision energy of 28.

The DIA data were searched against the human UniProt database using DIA‐NN. The search employed the trypsin/P digestion rule, ensuring high protein and peptide confidence levels, with a false discovery rate of .01. To enhance data comparability, post‐QC protein quantification data were normalised between samples using the ‘Aquantile’ method of ‘limma’^17^ package and log‐transformed for downstream analysis.

### 2.4 Dimensional reduction and differentially expressed proteins analysis

To visualise dimensional reduction, uniform manifold approximation and projection (UMAP) analysis was conducted using the ‘umap’ package.^18^ Differentially expressed proteins (DEPs) were identified using the ‘limma’ package,^17^ considering proteins with an absolute log fold‐change greater than 1 and an adjusted *p*‐value less than .05 as differentially expressed.

### 2.5 Enrichment analysis

All enrichment analyses were performed using the ‘clusterProfiler’ package.^19^ To elucidate the roles of tumour‐related proteins, we utilised the bioinformatics tool Metascape, which integrates data from Gene Ontology, Kyoto Encyclopedia of Genes and Genomes, Uniprot and DrugBank.^20^ For task #3, we applied fuzzy clustering using the ‘Mfuzz’ package before enrichment analysis to gain more explainable results.^21^, ^22^ This soft clustering algorithm differs from hard clustering in that it can assign samples to one or more clusters with a certain probability, making it particularly suitable for datasets that are difficult to distinguish and for reducing noise.

### 2.6 Feature selection

Flowchart of the feature selection of multitask protein panel is presented in Figure S2. The diagnostic efficacy of individual DEPs was evaluated using the area under the receiver operating characteristic (ROC) curves,^23^ which is a well‐established feature selection method.^24^, ^25^ For tasks #1 and #2, which involved dichotomised outcomes, the top 10 DEPs were selected. For task #3, a multi‐class classification, the top five DEPs were chosen based on various DEPs analyses.

Subsequently, selected proteins were subjected to least absolute shrinkage and selection operator (LASSO) regression to eliminate collinear proteins and prevent overfitting, utilising the ‘glmnet’ package.^26^ The optimal value of lambda was determined as the minimum value selected through 10‐fold cross‐validation.

### 2.7 Construction and validation of tumour‐related protein predictive model

Random forest model was implemented using the ‘randomforest’ package,^27^ with model constructed using 1000 trees and 10‐fold cross‐validation.^28^

Model validation was conducted using both internal and external datasets. For the internal validation set, block randomisation was performed with a 6:4 ratio. An independent external validation dataset comprised 46 patients from the TKLCM cohort.

Sensitivity and specificity of each task's protein panel were assessed using the ROC. The area under ROC (AUC) was used to quantify the performance, utilising the ‘pROC’ package for tasks #1 and #2^23^ and ‘MultiROC’ package for task #3.^29^ AUC was calculated with a 95% confidence interval.

### 2.8 Proteins feature scoring and model interpretation

Shapley additive explanations (SHAP) were employed to explain the random forest model base on the ‘fastshap’ package.^30^ SHAP is an explainable method derived from the game theoretic, specifically the Shapley value. Shapley value provide insights into how to distribute contribution among all proteins (players) within a model (coalition), based on the coalitional game theory. By applying this concept to machine learning predictive models, SHAP significantly enhances prediction transparency compared to other well‐known variable importance metrics.^31^, ^32^

## 3 RESULTS

### 3.1 Characteristics of patient

**FIGURE 1:**
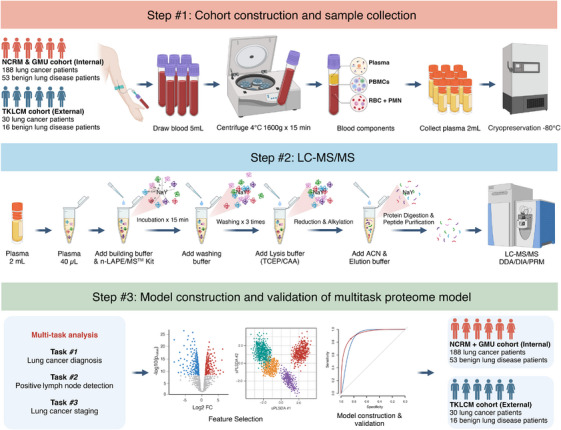
Schematic diagram of the study. Step #1: cohorts construction and sample collection. Step #2: liquid chromatography‒mass spectrometry/mass spectrometry (LC‐MS/MS). Step #3: model construction and validation of multitask proteome model. ACN, acetonitrile; CAA, 2‐chloroacetamide; n‐LAPE/MSTM, nano‐low abundant plasma enrichment/mass spectrometry TM; TCEP, tris (2‐carboxyethyl) phosphine hydrochloride).

A flowchart illustrating the research strategy is presented in Figure 1. In 1STGMU cohort, the mean age of the patients was 61.11 years (range 37‒81 years), with 83 (65.35%) being male. Of the patients, 46 had a history of hypertension, 34 had diabetes, 19 had cardiovascular disease (CVD), one had arrhythmia and one had chronic obstructive pulmonary disease. In NCRM cohort, the mean age of the patients was 61.28 years (range 18‒85 years), with 70 (69.31%) being male. Of the patients, 32 had a history of hypertension, 23 had diabetes, 10 had CVD, one had arrhythmia and one had a history left parotid gland tumour, which had been surgically resected. In TKLCM cohort, the average age of the patients was 40.3 years (range 25‒71 years), with 23 (50%) being male. No adverse events were observed in any enrolled patients. Baseline characteristic is shown in Table S1.

The results of UMAP dimensionality reduction, which distinguished lung cancer patients and benign lung disease patients based on different underlying conditions, indicated no significant differences in either group (Figure S6A,B). The UMAP dimensionality reduction results for patients categorised as normal and overweight based on the BMI also showed no significant differences (Figure S6C).

### 3.2 NaY‐based plasma proteomics identifying target population in multiple diagnostic tasks

After QC and quantitative, a total of 4703 plasma protein quantification data were utilised. The evaluation results of the QC are shown in Supporting Information S4. To balance quantification from different cohort, we applied quantile normalisation, which ensured consistent statistical properties, enabling accurate cohort comparisons. Subsequent analyses were conducted separately to account for any residual cohort‐specific differences, confirming the cohort's comparability and enhancing the validity of our findings. The homogeneity of protein detection in the raw data allowed for robust downstream analysis (Figure S2).

**FIGURE 2:**
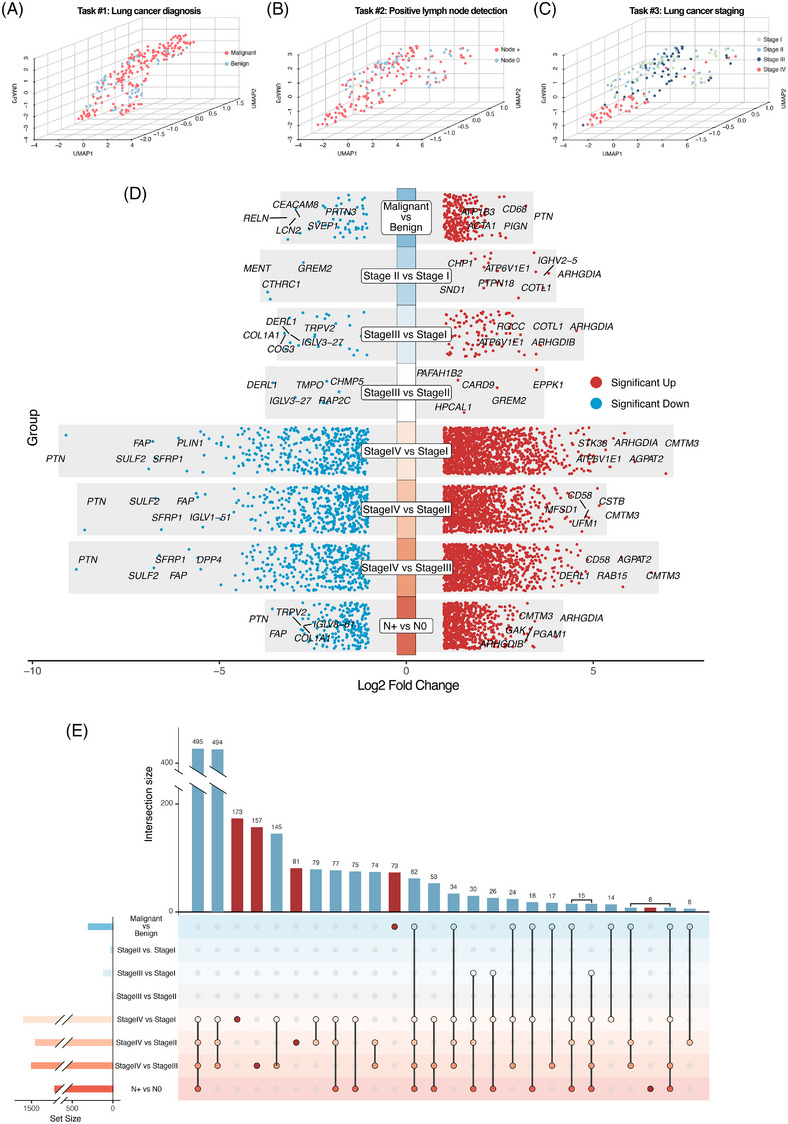
Dimension reduction and differentially expressed proteins analysis. (A‒C) The uniform manifold approximation and projection (UMAP) visualisation presented a three‐dimensional unsupervised embedding of high‐dimensional proteomic data for each sample, each point represented a sample and was colored based on its classification task. (D) Results of differential expression proteins (DEPs) analysis in different groups, represented by the log2 fold change in gene expression. Red dots indicating significantly upregulated genes (*p* < .05) and blue dots indicating significantly downregulated genes (*p* < .05). (E) Upset plot diagram of the DEPs analysis results and shared DEPs in different comparisons. The connected dots below the bar plot illustrated the specific comparisons involved in each intersection. The bar plot at the top shows the intersection size, while the horizontal bars on the left depict the set size, representing the total number of DEPs in each comparison.

To assess the ability of proteomic data to distinguish between different groups, we performed dimensional reduction using UMAP analysis. Consistent with previous studies, the plasma proteome exhibits dynamic changes during tumourigenesis and progression. NaY‐based proteomics showed a capability in distinguishing benign from malignant patients (task #1; Figure 2A). For tumour staging (task #2; Figure 2B) and predicting lymph node metastasis status (task #3; Figure 2C) among malignant patients, NaY‐based proteomics also showed its effectiveness, particularly in detecting patients in stage IV.

We then conducted differential protein analysis to identify candidate proteins for further enrichment analysis and development of predictive models. Among all assayed proteins, 300 were identified as DEPs for task #1 including 255 significantly upregulated proteins and 45 significantly downregulated proteins, and 920 for task #2 including 716 significantly upregulated proteins and 204 significantly downregulated proteins (Figure 2D). For task #3, a total of 2251 proteins were identified as DEPs. Notably, the majority of DEPs were shared among two or more comparison groups, while only a small subset unique to individual comparison groups (Figure 2E).

### 3.3 NaY‐based plasma proteomics reveals biological event heterogeneity during lung cancer progression

**FIGURE 3:**
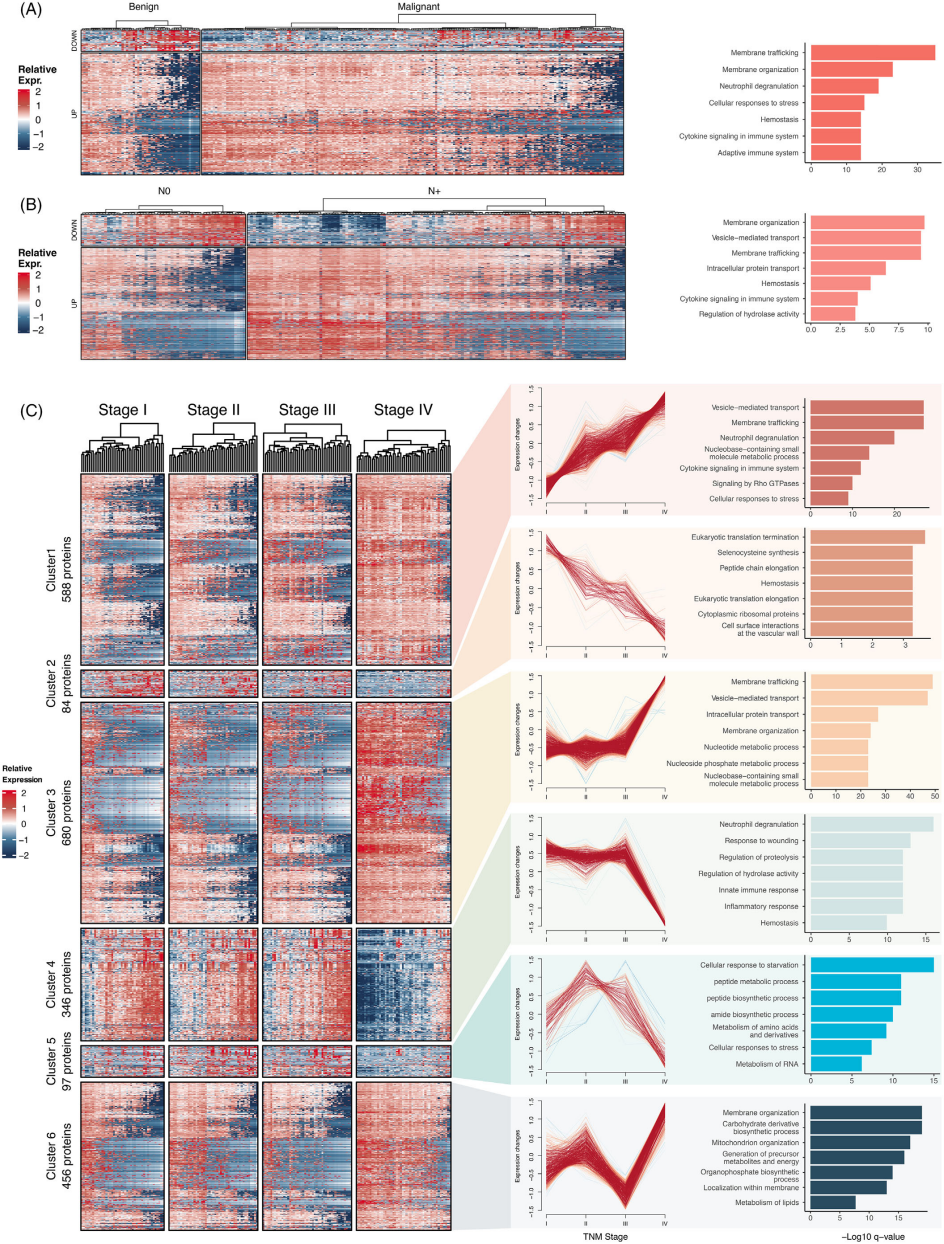
Functional enrichment of differentially expressed proteins. Heatmaps compared gene expression patterns in benign and malignant samples (A) and lymph node metastasis and non‐metastasis samples (B), colours represented the relative expression levels of each differentially expressed protein (DEP), with red indicted upregulation and blue indicated downregulation. Bar chart on the right illustrated the top biological processes that DEPs significantly enriched. (C) Illustration of DEPs clusters across lung cancer stages (stage I to IV). Expression levels of proteins in each DEP cluster were visualised using heatmap. The line plots showed the expression changes across stages for each DEP cluster. The bar chart on the right illustrated the top biological processes in which each DEP cluster was significantly enriched.

To interpret the function of DEPs in plasma, enrichment analysis of DEPs was performed using Metascape. The DEPs identified in task #1 are closely associated with cellular membranes homeostasis events such as membrane trafficking and membrane organisation. Additionally, proteins associated with immune response (neutrophil degranulation), cellular stress and coagulation events also differential expressed (Figure 3A). DEPs identified in task #2 reflects the alterations of plasma in patients with lymph node metastasis, which can be enriched in biological events mainly about membrane homeostasis, protein transport and cytokine signalling (Figure 3B).

For DEPs identified in task #3, we performed an unsupervised cluster analysis using the fuzzy clustering and identified six distinct protein expression patterns during tumour progression (Figure 3C). Clusters 1 and 2 contained 672 DEPs and showed monotonic changes from stage I to IV. Expression levels of DEPs in cluster 1 continuously increased. DEPs in cluster 1 can be also enriched in cellular membranes homeostasis‐related pathways like vesicle‐mediated transport and events related to immune response like cytokine signalling. DEPs in cluster 2 were downregulated during tumour progression. They can be enriched in biological events associated with peptide translation and haemostasis. Notably, the plasma DEPs in clusters 1 and 2 exhibited overlap with the DEPs identified in tasks #1 and #2 mentioned above. This may be a result of the shared biological mechanisms in tumour initiation and progression.

Clusters 3 and 4 contained 1026 DEPs and exhibited drastic changes at in the advanced stage (stage IV), instead of stages I‒III. DEPs that only highly expressed in stage IV (cluster 3) were also related to cellular membrane system homeostasis and nucleotide metabolic process, while DEPs specifically downregulated in stage IV (cluster 4) reflect addition dysregulation in immune and metabolic processes in high‐grade tumours. In detailed, DEPs in cluster 4 were related to neutrophil degranulation, response to wounding, regulation of proteolysis, regulation of hydrolase activity, regulation of hydrolase activity and innate immune response.

Besides the DEP clusters mentioned above, clusters 5 and 6 revealed that NaY‐based proteomics can sensitively detect other subtle dynamic alternations across tumour stages. DEPs in cluster 5 had an inverted U‐shape expression pattern, enriched in pathways like peptide biosynthetic process and amino acid metabolism. In cluster 6, DEPs can be enriched in pathways like membrane organisation and energy metabolism. This highlights the advantage of NaY‐based plasma proteome in offering insights into tumour progression across all stages.

For each cluster, we validated the protein‒protein interaction (PPI) patterns and identified the hub proteins within the PPI network of each cluster (Figure S4).

We further performed an intersection analysis on all DEPs, the results showed that a total of 111 proteins exhibited co‐expression patterns across the DEP analyses of different tasks (Figure S5A). Further enrichment results showed that multiple pathways and functional modules were significantly enriched in the target gene set, including membrane organisation, vesicle‐mediated transport and intracellular protein transport. The significant enrichment of these processes suggests that the target genes play essential roles in cell membrane stability, vesicle transport and protein translocation, which may be crucial for maintaining cellular structure and material transport balance. Meanwhile, several signalling pathways were also significantly enriched, such as the mTOR signalling pathway and the Fas ligand pathway and heat shock protein stress induction, which play key roles in regulation of cell growth, stress response and apoptosis (Figure S5B).

In all, the NaY‐based proteomic elucidated the biological heterogeneity of plasma proteins across various stages of tumour progression. Notably, biological events pertaining to the homeostasis of cytoplasmic and organelle membranes, immune responses and metabolic processes were recurrently observed, highlighting their critical roles.

### 3.4 Protein panel #1 enables effective non‐invasive screening of lung cancer (task #1)

**FIGURE 4:**
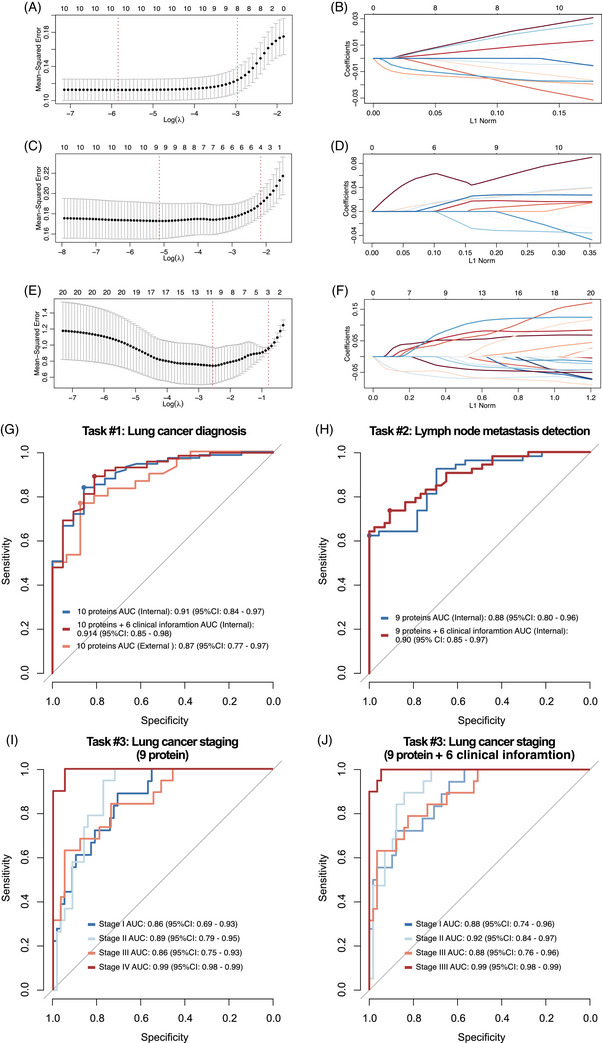
Feature selection, construction and validation of multitask predictive model. (A) Plots for least absolute shrinkage and selection operator (LASSO) regression coefficients for task #1. (B) Cross‐validation plot for the penalty term for task #1. (C) Plots for LASSO regression coefficients for task #2. (D) Cross‐validation plot for the penalty term for task #2. (E) Plots for LASSO regression coefficients for task #3. (F) Cross‐validation plot for the penalty term for task #3. (G) Receiver operating characteristic (ROC) curve of 10 proteins panel predictive model for task #1. (H) ROC curve of nine protein panel predictive model for task #2. (I) ROC curve of nine protein panel predictive model for task #3. (J) ROC curve of nine protein combined six clinical factors panel predictive model for task #3.

DEPs identified in each task were adopted for following model construction. We firstly assessed the prognostic potential of each protein individually by employing ROC curves to evaluate the ability of single protein expression levels to predict clinical outcomes. Among them, top 10 proteins were selected to develop a multi‐protein predictive model. To reduce multicollinearity, we performed LASSO regression, which yielded a 10 proteins (BAK1, CTSW, MICOS13, RELN, PTN, TPT1, SVEP1, PDGFD, LCN2 and GLG1) as protein panel #1 for construction and validation for task #1 model (Figure 4A,B).

In the internal validation cohort, the predictive AUC was .91 (.84‒.97), while .87 (.77‒.97) in the external validation. We further investigated whether the integration of the NaY‐based proteomics panel with clinical information could enhance diagnostic efficacy. After adding six clinical factors (age, gender, smoking history, CEA, CA‐125 and CA‐153) into prediction model, a significant increase of the AUC is observed in the internal validation: .91 (.85‒.98) (Figure 4G).

To further validate the proteins selected in panel #1, 102 healthy control participants were recruited from the TKLCM cohort. Of these participants, 20 were male and 82 were female, and 99 participants were of Han Chinese ethnicity. The mean age was 54.14 ± 7.64 years. Detailed baseline information was provided in Table S2. PCA indicated that there were no significant differences in proteins expression profiles between the benign lung disease patients from the GMU and TKLCM cohorts and healthy controls cohorts (Figure S7A). We subsequently conducted a DEP analysis of plasma protein expression levels measured in lung cancer patients and healthy controls, comparing the results with those obtained from the DEP analysis of lung cancer patients and benign lung diseases. Results revealed that 66.78% of upregulated DEPs overlapped between the two analyses, while 10.85% of downregulated DEPs overlapped. Among the 10 proteins in the protein panel #1, nine exhibited consistent expression patterns between patients with benign lung diseases and healthy controls. Notably, only LCN2 showed differential expression between these two groups (Figure S7B,C). To further validated the predictive performance of the proteins selected in protein panel #1 for distinguishing between lung cancer patients and healthy controls, we proceeded to use the 10 proteins identified in this study for distinguishing between benign and malignant patients (panel #1) to build a predictive model using the random forest method with 10‐fold cross validation. Result demonstrated that the proteins in the protein panel #1 effectively distinguished lung cancer patients from healthy controls, achieving an AUC of .98 (.96‒.99) (Figure S7D).

### 3.5 Protein panel #2 enables effective non‐invasive predicting lymph node metastasis (task #2)

Similar to task #1, we filtered for top 10 DEPs with the highest AUC in predicting lymph node metastasis. After applying LASSO regression, a panel of nine proteins (XPO1, NPNT, SND1, ARHGDIB, RELCH, PDLIM1, PRKG1, MAPK14, SPARC) as protein panel #2 was left for task #2 model construction and validation (Figure 4C,D).

In the testing set, the predictive AUC was .88 (.80‒.96). Integrated model of nine protein panel with six clinical factors, increase diagnostic accuracy was observed with AUC of .90 (.85‒.97) (Figure 4H).

### 3.6 Protein panel #3 enables effective non‐invasive predicting TNM stage (task #3)

The top five proteins with the highest AUC values from 2251 identified DEPs in comparisons between any two stages were selected, leaving 20 proteins. After LASSO regression, 10 proteins (KPNB1, COTL1, XPO1, PTN, TARBP1, DERL1, CARD9, SULF2, PF4, ARHGEF2) were finally used as protein panel #3 for model construction (Figure 4E,F).

In the internal validation cohort, the predictive AUC was .86 (.69‒.93) for stage I, .89 (.79‒.95) for stage II, .86 (.75‒.93) for stage III and .86 (.75‒.93) for stage IV (Figure 4I). Incorporating six clinical factors increased the AUC in validation, stage I had an AUC of .88 (.74‒.96), stage II had an AUC of .92 (.84‒.97), stage III had an AUC of .88 (.76‒.96) and stage IV had an AUC of .99 (.98‒.99) (Figure 4J).

### 3.7 Proteomics diagnostic models reveal biologically significant predictors across tasks

**FIGURE 5:**
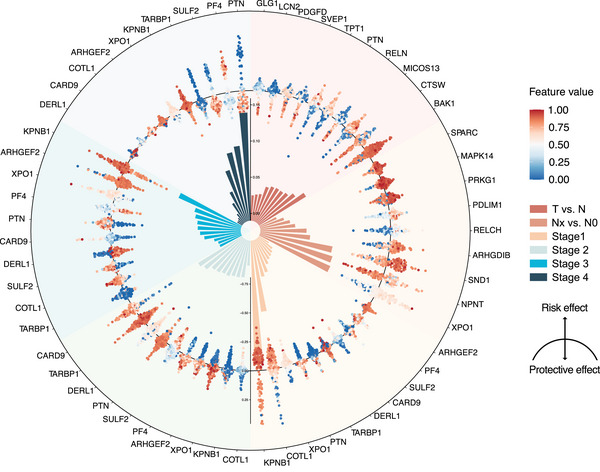
Shapley additive explanations (SHAP) values for multitask model. Inner loop showed the average absolute SHAP value for each protein. The bar represented the extent of the protein's contribution to the outcomes associated with each diagnostic task, the higher the bar, the greater the contribution. The colour of the swarm plot indicated the amount of plasma protein, ranging from low (blue) to high (red), as shown by the colour bar. Deviations towards the centre and periphery represented protective and risk contributions, respectively.

Protein panel for each task is detailed in Table S3. We used SHAP method to identify of the most significant discriminators for each task and clarified whether each outcome represented a risk factor or a protective factor in every model. For task #1, the protective factors identified were CTSW, RELN and SVEP1, while BAK1, MICOS13 and PTN were identified as risk factors. In task #2, NPNT emerged as a protective factor, whereas XPO1, SND1 and ARHGDIB were classified as risk factors. Task #3, involving the identification of lung cancer stages, revealed stage‐specific proteins. In stage I, biomarkers COTL1, XPO1 and CARD9 were the strongest indicative biomarkers, while KPNB1, PTN and TARBP1 suggested a lower likelihood of stage I. For stage II, DERL1, SULF2 and COTL1 were characteristic biomarkers, whereas CARD9, TARBP1 and PTN indicated it was less likely to be stage II. In stage III, biomarkers such as ARHGEF2, PF4 and DERL1 were defining, while KPNB1, XPO1 and PTN suggested a lower likelihood of stage III diagnosis. For stage IV, PTN, SULF2 and TARBP1 were characteristic biomarkers, but PF4, KPNB1 and XPO1 implied a lower likelihood of stage IV diagnosis (Figure 5).

These biomarkers are often proteins that have been proven to be closely related to tumour progression. For example, the strongest risk factors in task 1 were BAK1, MICOS13 and PTN. BAK1, a pro‐apoptotic protein, regulates cell death, possibly related to the survival of cancer cells.^33^, ^34^ MICOS13, involved in mitochondrial structure, affects metabolism and apoptosis, linking it to cancer progression.^35^ PTN promotes cell growth, angiogenesis and metastasis.^36^, ^37^, ^38^ This suggests that the dynamic evolution of proteins occurs not only in the tumour but also in peripheral plasma. Although the mechanisms require further research, this indicates the immense potential of plasma proteomics.

## 4 DISCUSSION

Dynamic monitoring of lung cancer events at multiple time points is an essential for prevention, diagnosis and treatment.^39^ In this study, we prospectively enrolled 287 patients from three centres in China. Using plasma samples co‐incubated with a novel nano adsorbent, zeolite NaY,^16^ and leveraging data from high throughput proteomic profiling, we conducted a comprehensive multitask proteomic analysis. Ultimately, single‐digit level protein panels for each respective task were ultimately filtered and showed a high‐performance diagnostic ability. Most of selected proteins have not been previously identified, mainly due to the enhanced depth of MS and improved detection of low‐abundance proteins achieved using NaY. Acknowledging acceptable variations in plasma protein expression under different baseline conditions, which reflect the diverse characteristics of patients in real‐world lung cancer screening, we found that our selected proteins maintained strong predictive performance across various outcomes. This supported their utility as non‐invasive biomarkers applicable to heterogeneous clinical populations.

With the increasing focus on proteomics, several studies have highlighted the potential of protein biomarker in the field of lung cancer.^2^, ^3^, ^40^, ^41^, ^42^ The main objective of these studies was to differentiate between benign and malignant, which aligns with the task #1 in our research. In comparison, our model demonstrates greater accuracy, reasonable, efficiency and requires fewer proteins than previous research. Although consensus has been achieved that non‐invasive biomarker plays an important role in whole‐time cancer monitoring and assessment,^39^ previous studies have not extensively focused on their utility in staging (task #3) and predicting lymph node metastasis (task #2). Our study developed a multitask predictive model that demonstrated high accuracy and reliability. In stage prediction, a multi‐label classification problem, our results showed a robust ability in discriminating between each stage. Notably, our model exhibited excellent discriminatory capability for advanced lung cancer, achieving an AUC of .99.

Based on the multitask predictive model, a total of 27 proteins were selected as the final modelling proteins for the tree tasks. These proteins not only played critical biological roles in the initiation and progression of lung cancer, previous studies have also demonstrated that they exert significant effects in various other cancer types. Recent studies have elucidated the roles of various proteins in cancer progression and potential therapeutic targets. Jones et al.^43^ demonstrated that BAK, a pro‐apoptotic member of the BCL2 family, mediates apoptosis by interacting with MCL‐1 and BCL‐xL; upon activation by BH3‐only proteins, BAK is released to induce cell death, highlighting its potential as a therapeutic target. Nomura and Katunuma^44^ found that CTSW, a member of the cathepsin protease family, is closely associated with tumour invasion, metastasis and proliferation by degrading the extracellular matrix and basement membrane. Qin et al.^45^ reported that RELN enhances tumour cell proliferation and glycolysis in multiple myeloma by promoting the Warburg effect, identifying downstream Akt and STAT3 signalling pathways as potential therapeutic targets. Papadimitriou et al.^46^ showed that PTN, a secreted heparin‐binding growth factor, regulates angiogenesis and cancer progression by binding to receptor protein tyrosine phosphatase beta/zeta; both PTN and RPTPβ/ζ are overexpressed in various cancers, promoting tumour growth and invasion. Bae et al.^47^ indicated that TPT1 is highly expressed in tumour cells and inhibits autophagy by modulating the MTORC1 and AMPK pathways; loss of TPT1 enhances autophagy and affects the BECN1‐mediated protein network, influencing tumour cell activity. Amson et al.^48^ identified TPT1 as a potential prognostic marker in breast cancer, emphasising its role in regulating the p53 signalling pathway and reprogramming tumour cell phenotypes, acting as a critical regulator of cancer stem cell function and tumour reversal. Wu et al.^49^ reported that the long non‐coding RNA TPT1‐AS1 activates TPT1 through the PI3K/AKT signalling pathway, promoting ovarian cancer proliferation and metastasis, with high expression levels correlated with adverse pathological features. Shih and Holland^50^ demonstrated that PDGF‐D promotes tumour proliferation and metastasis by modulating angiogenesis and stromal remodelling within the tumour microenvironment, associated with the development of central nervous system tumours such as gliomas. Santiago‐Sánchez et al.^51^ found that LCN2, a secreted glycoprotein, facilitates tumour cell proliferation, angiogenesis, invasion and metastasis by regulating the activity of matrix metalloproteinase‐9, suggesting its potential as a therapeutic target. Antonenko et al.^52^ identified GLG1, a Golgi‐associated protein functioning as a selectin ligand, as a potential interaction partner of the Bcr‐Abl oncoprotein in chronic myeloid leukemia; its phosphorylation may influence disease progression by altering Golgi function and associated signalling pathways. Trkulja et al.^53^ reported that XPO1 is highly expressed in non‐Hodgkin's lymphoma, and its inhibitor, selinexor, demonstrated significant anticancer efficacy, particularly in relapsed or refractory patients unresponsive to chemotherapy. Jariwala et al.^54^ indicated that SND1 is overexpressed in various cancers, including breast, prostate, colorectal cancer and malignant gliomas, regulating multiple mechanisms of gene expression such as transcriptional activation, RNA stability and RNA interference. Zhou et al.^55^ showed that PDLIM1, a member of the PDZ‐LIM family functioning as a cytoskeletal protein and signalling molecule, is dysregulated in various cancers and is associated with tumourigenesis and progression. Islam et al.^56^ found that reduced expression of PRKG1 is associated with colorectal cancer progression; activation of PRKG1 decreases tumour cell proliferation and invasion while inducing apoptosis. Wu et al.^57^ further demonstrated that PRKG1 inhibits the epidermal growth factor‐induced MAPK/ERK signalling pathway, reducing the invasive and proliferative capabilities of gastric cancer cells, suggesting PRKG1 as a potential therapeutic target. Liu et al.^58^ reported that MAPK14 is highly active in clear cell renal cell carcinoma, promoting tumour proliferation and migration by regulating the cell division cycle protein CDC25B; inhibiting MAPK14 significantly reduces tumour cell proliferation and migration. XPO1, a nuclear export protein responsible for transporting tumour suppressor proteins such as p53 and RB from the nucleus to the cytoplasm, is overexpressed in various solid tumours and haematologic malignancies, promoting cancer cell proliferation and survival.^59^ Tan et al.^60^ reported that Derlin‐1 is overexpressed in colorectal cancer tissues and is associated with Dukes staging, lymph node and distant metastases, and poor prognosis; inhibition of the PI3K/AKT signalling pathway can reduce cancer cell proliferation. Tao et al.^61^ demonstrated that SULF2 promotes proliferation and metastasis of colorectal cancer by activating the Akt and Erk1/2 signalling pathways; its high expression is associated with malignant clinical features and poor prognosis, and knockdown of SULF2 significantly suppresses tumour growth and invasion.

The innovation and commercialisation of high‐throughput sequencing technology have significantly advanced the study of biomarkers, including genomics, transcriptomics and proteomics.^62^, ^63^, ^64^, ^65^ Among these, proteomics stands out due to the extensive and complex composition of proteins, offering deeper and broader data characteristics and relatively independent metabolic pathways.^66^, ^67^ Proteins, being the direct executors of life activities, are particularly suitable as biomarkers for indicating abnormalities in the physiological and pathophysiological processes, which have unique advantages in identifying disease‐related protein molecules, discovering tumour markers and therapeutic targets.^68^, ^69^

From an economic perspective, cost of MS analysis and protein kit is often lower compared to other types of biomarkers. In certain clinical laboratory setting, the cost for small molecule analysis can be as low as one US dollar per sample, this is of particular interest for a future translation of the results into clinical application and screening in large population.^70^

Despite the community with a strong sense of self‐efficacy are full of confidence, the discovery, validation and clinical application of new biomarkers become difficult.^71^, ^72^ Innovation technology could build a continuous biology pipeline.

Previous studies noted the difficulties encountered by finding and validating new biomarker use proteomics, which could be partly explained by the masking effect caused by high‐abundance proteins, which could hinder the identification of low‐abundance proteins. Using particles could solve this problem, particles in the plasma could adsorb proteins as ‘protein corona’^73^ When adsorbed onto the surface of nanoparticles, protein corona can act as a ‘concentrator’ of plasma proteins, enhancing the detection of low‐abundance proteins.^74^, ^75^ Blume et al.^76^ developed a maturation platform using multiple magnetic nanoparticles and the protein corona, which can detect approximately 2000 proteins, thereby improved plasma proteome coverage. However, this methodology requires separate co‐incubation of nanoparticles with the plasma sample and multiple assays with liquid chromatography‒mass spectrometry/mass spectrometry (LC‐MS/MS), resulting in considerable time and expense.

In our study, we used a type of adsorbent, zeolite NaY, which previously reported by our research team. Previous study published by our team systematically compared NaY plasma protein corona and traditional plasma proteomics.^16^ Three methods for processing plasma samples in proteomics analysis, direct enzymatic digestion and analysis, high‐abundance protein depletion using the top 14 methods, and the plasma protein corona strategy based on the zeolite NaY. Results showed that compared with the commercial top 14 methods and traditional plasma proteomics, zeolite NaY‐based proteomics has a significant advantage in enriching plasma proteins.

Zeolite NaY has demonstrated outstanding performance in gas and heavy metal ion separation and adsorption.^77^ Zeolite NaY interacts strongly with protein molecules containing polar groups due to the strong polarity of its cavities.^78^ The surface micropores of zeolite significantly influence protein‒zeolite interactions, recognising certain amino acid residues and size‐matched protein secondary structures, thereby reducing adsorption free energy and enhancing interactions.^79^ Additionally, the negative surface charge, surface silica hydroxyl groups and adjustable hydrophobicity allow zeolites to interact with proteins through van der Waals forces, electrostatic interactions^80^ and hydrogen bonding.^81^ These combined properties enable zeolites to capture a broader range of proteins, suggesting that zeolite NaY has great potential in addressing the masking effect caused by high‐abundance proteins during plasma proteome analysis. This viewpoint was also demonstrated in our previous study, where a comparison of the top 20 high‐abundance proteins between the NaY‐based plasma group and the traditional plasma group revealed this effect. Results showed that the top 20 most abundant proteins in plasma exhibit a significant reduction in abundance after NaY enrichment. Moreover, the top 20 proteins with the highest abundance after NaY enrichment are not the same as the top 20 most abundant proteins in the original plasma, further demonstrated NaY's capability in removing high‐abundance proteins.^16^

Several potential shortcomings in the study need to be acknowledge. First, although generating promising results and demonstrating the robust in an independent cohort, establishing interlaboratory standardised operating procedures for this analytical process may prove challenging due to the complex and variable methodological stages. Second, our study exclusively included Chinese participants, limiting the generalisability of the findings to other populations. Although we attempted to validate our findings using plasma protein data measured by Olink technology in the UK‐Biobank, the effort was unsuccessful due to inadequate overlap between our study and the UK‐Biobank data. Third, further studies with larger cohorts are necessary to validate our predictive proteomic classification.

## 5 CONCLUSIONS

In summary, leveraging the novel proteomics approach, we have developed a highly accurate multitask predictive model for lung cancer. this plasma‐based predictive measure showed significant potential for clinical diagnosis and future treatment management. Certainly, these predictive panels must be further refined, simplified and rigorously validated in other independent cohorts, and efforts should be made to translate these panels into a simpler, automated platform suitable for border application.

## AUTHOR CONTRIBUTIONS

*Conceptualisation*: Wenhua Liang, Jun Liu and Jiangxing He. *Data curation*: Hengrui Liang, Runchen Wang, Ran Cheng, Na Zhao, Zhanpeng Jiang and Yun Yan. *Formal analysis*: Hengrui Liang, Runchen Wang and Zhiming Ye. *Funding acquisition and investigation*: Ying Huang, Wangzhong Li and Xin Xu. *Methodology*: Runchen Wang, Zhiming Ye, Na Zhao and Lei Song. *Project administration*: Hengrui Liang and Runchen Wang. *Resources*: Xiaohong Zhao, Jie Li and Jianxing He. *Software*: Runchen Wang, Jianqi Zheng and Wenhua Liang. *Supervision*: Hengrui Liang, Wenhua Liang, Jie Li, Jun Liu and Jianxing He. *Validation*: Xiaohong Zhao, Ying Huang and Wenhua Liang. *Visualisation*: Runchen Wang, Hongsheng Deng and Yu Jiang. *Writing—original draft*: Hengrui Liang, Runchen Wang and Zhiming Ye. *Writing—review and editing*: Yuechun Lin, Zhanpeng Jiang and Jianxing He. All author contributed to the design, data analysis and results interpretations. The corresponding authors had full access to the data in the study and takes responsibility for the integrity of the data and the accuracy of the data analysis.

## CONFLICT OF INTEREST STATEMENT

The authors declare no conflicts of interest.

## ETHICS STATEMENT

This prospective study was approved by the ethics committee of NCRM and 1STGMU (approval number: ES‐2024‐051‐02) and was registered with clinical trial registry (clinical trial number: NCT06422637). This study was conducted in accordance with the Declaration of Helsinki. All the patients newly diagnosed with lung cancer or benign lung disease were routinely asked to donate blood samples for research purpose, with both verbal and written informed consent obtained. This study followed the Strengthening the Reporting of Observational Studies in Epidemiology (STROBE) reporting guideline for cohort studies.

## CONSENT FOR PUBLICATION

All authors read and approved the final version of the manuscript.

## Supporting information



Supporting information

Supporting information

Supporting information

Supporting information

Supporting information

Supporting information

Supporting information

Supporting information

Supporting information

Supporting information

Supporting information

Supporting information

Supporting information

Supporting information

## Data Availability

The datasets used in this study are available from the corresponding author on a reasonable request. Patient data are not publicly available because the contain sensitive information that could compromise patient privacy.
