# Supplementary figures and images for: LcProt: Proteomics‐based identification of plasma biomarkers for lung cancer multievent, a multicentre study
Source: Clin Transl Med. 2025 Jan 9;15(1):e70160. doi: 10.1002/ctm2.70160 (PMC11714244; doi:10.1002/ctm2.70160)

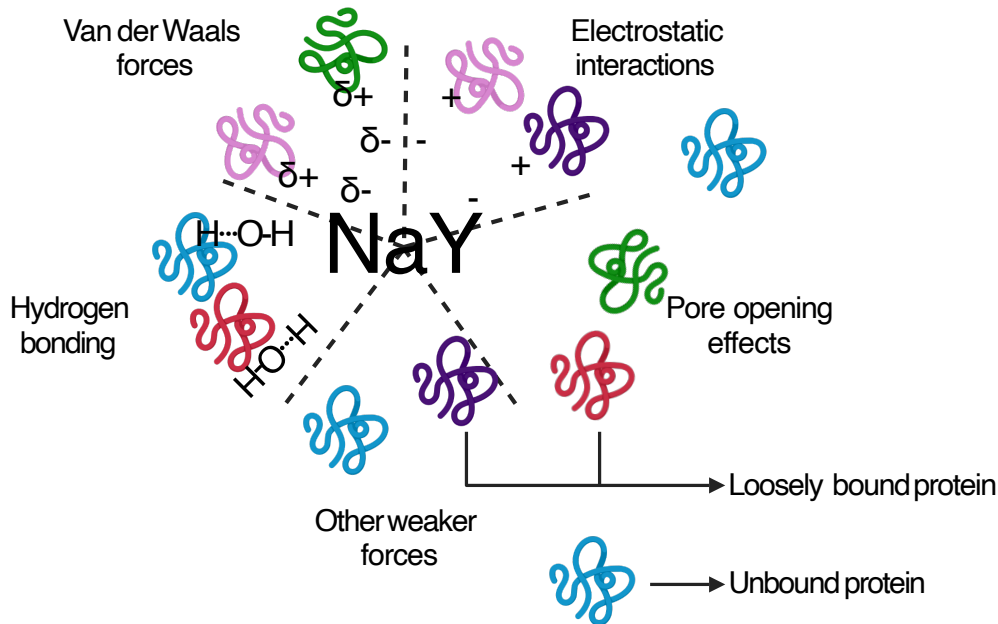

Supplement: Supplementary file 1 — Supporting information [file CTM2-15-e70160-s003.pdf]

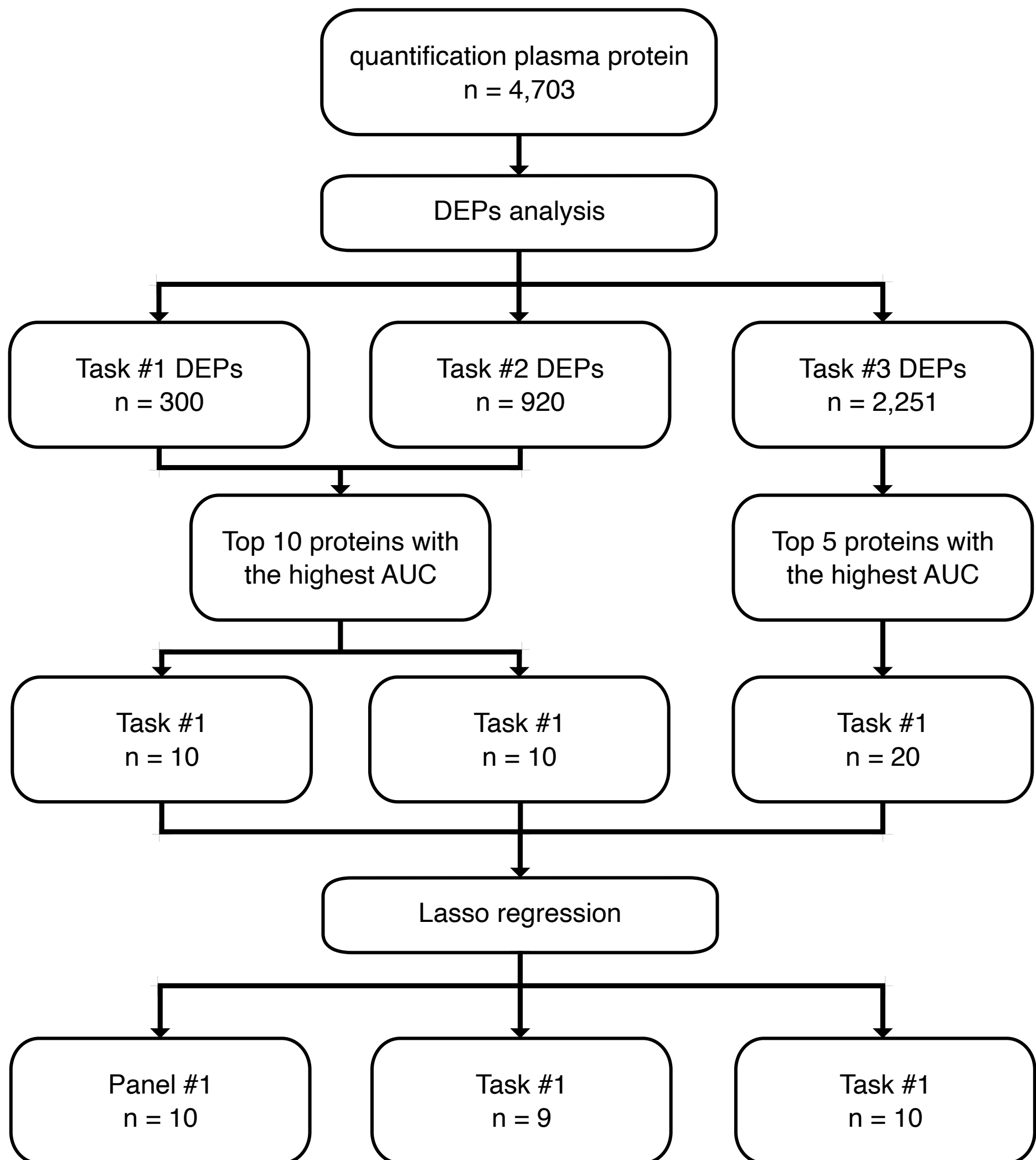

Supplement: Supplementary file 2 — Supporting information [file CTM2-15-e70160-s005.pdf]

A

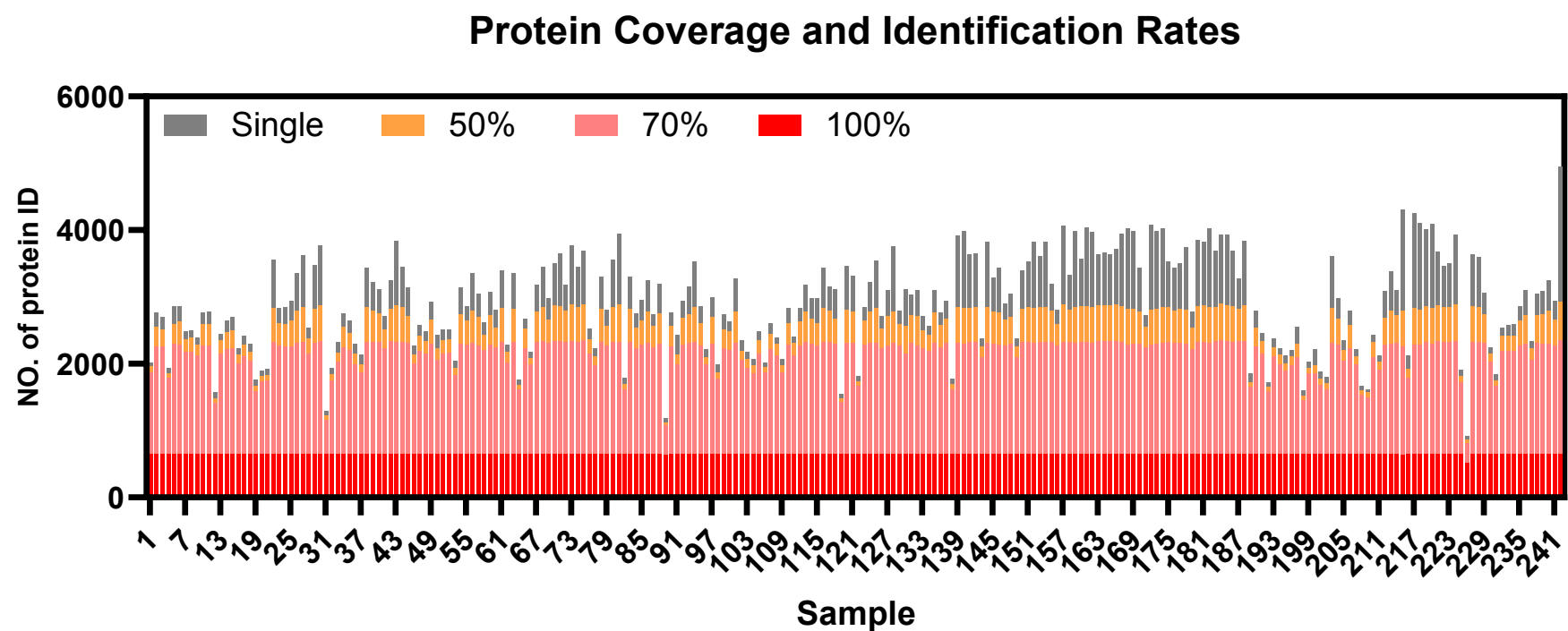

B

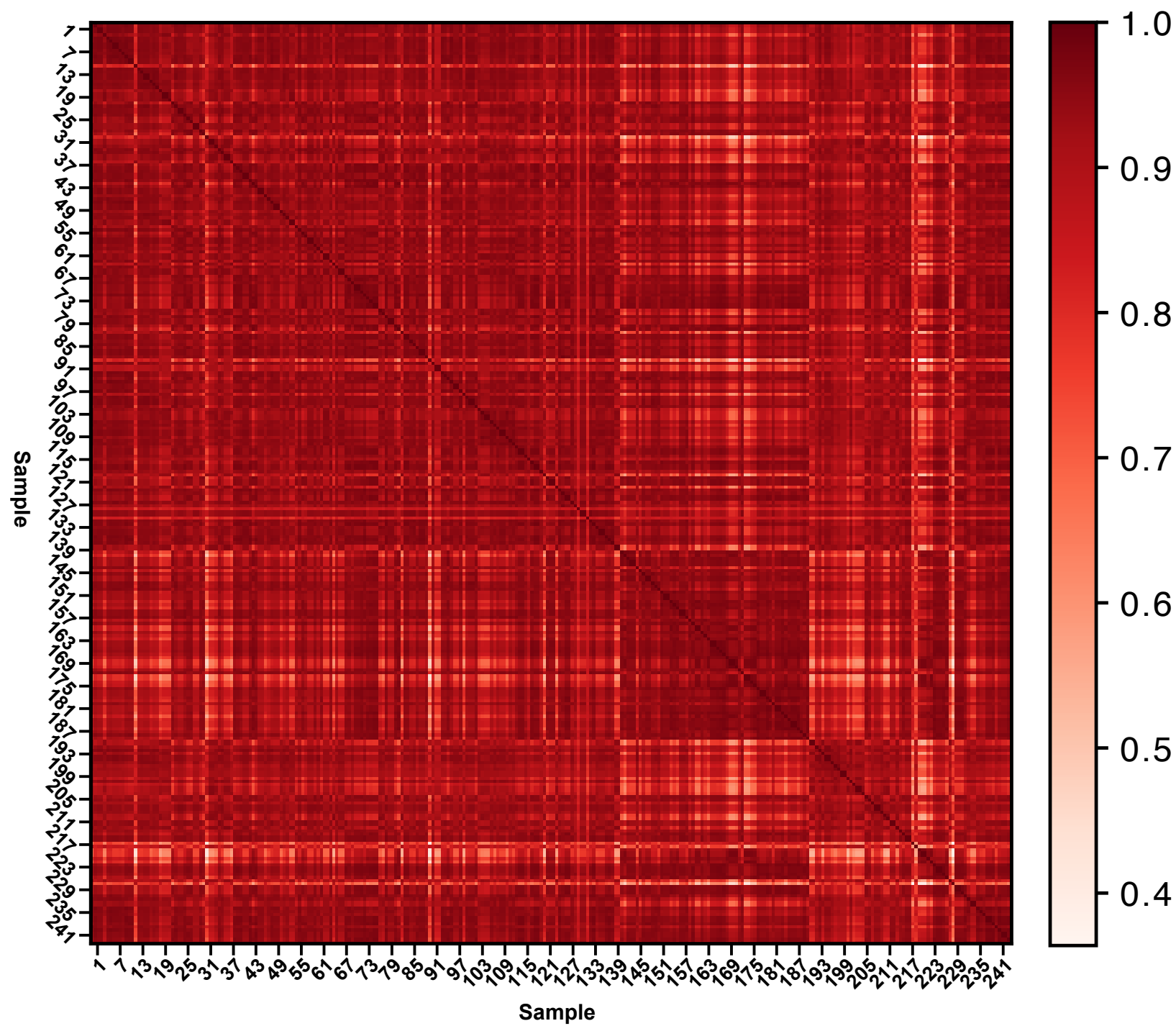

Supplement: Supplementary file 3 — Supporting information [file CTM2-15-e70160-s013.pdf]

A

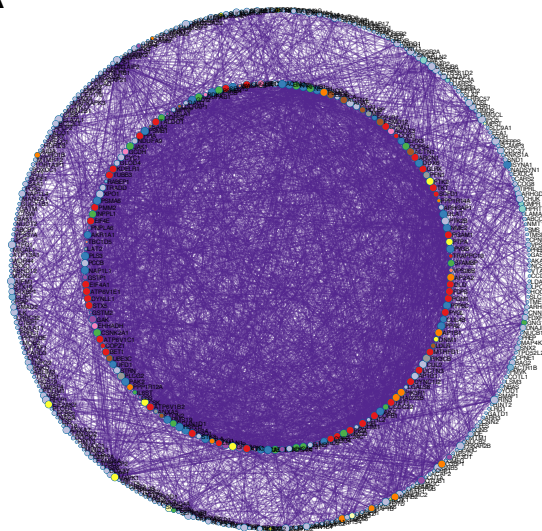

B

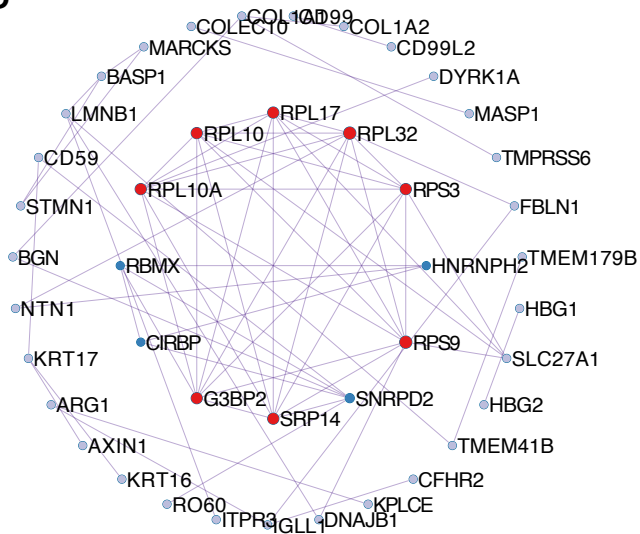

C

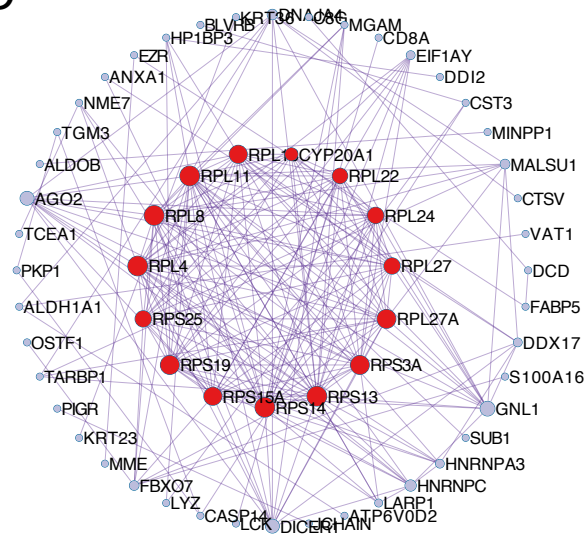

C

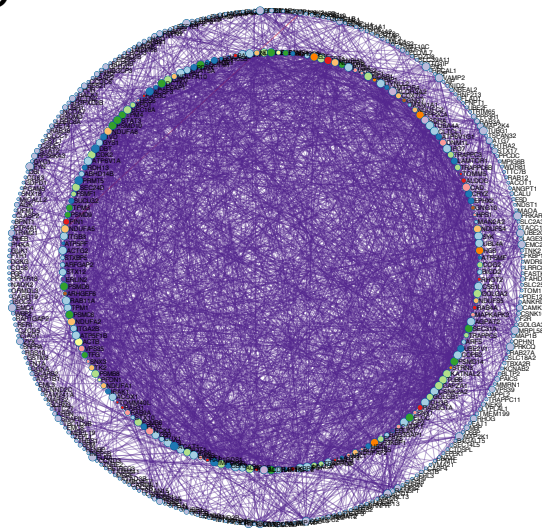

D

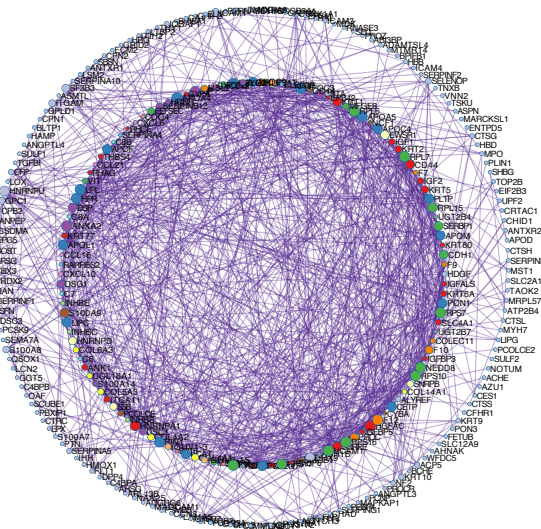

E

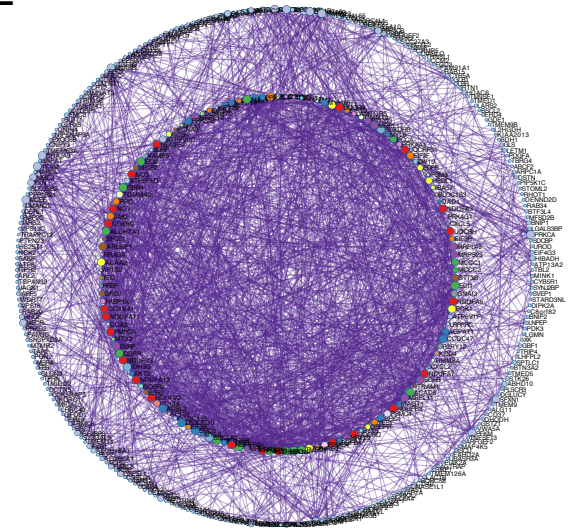

Supplement: Supplementary file 4 — Supporting information [file CTM2-15-e70160-s009.pdf]

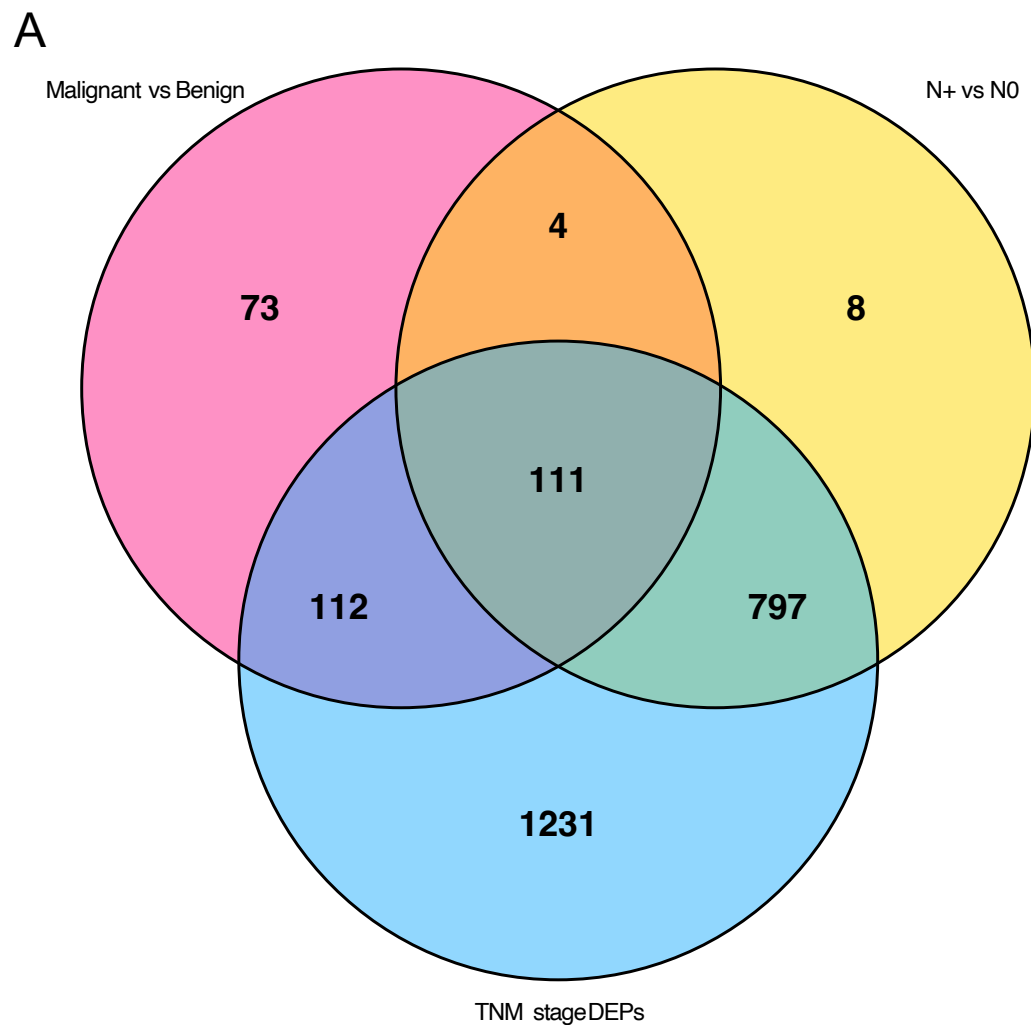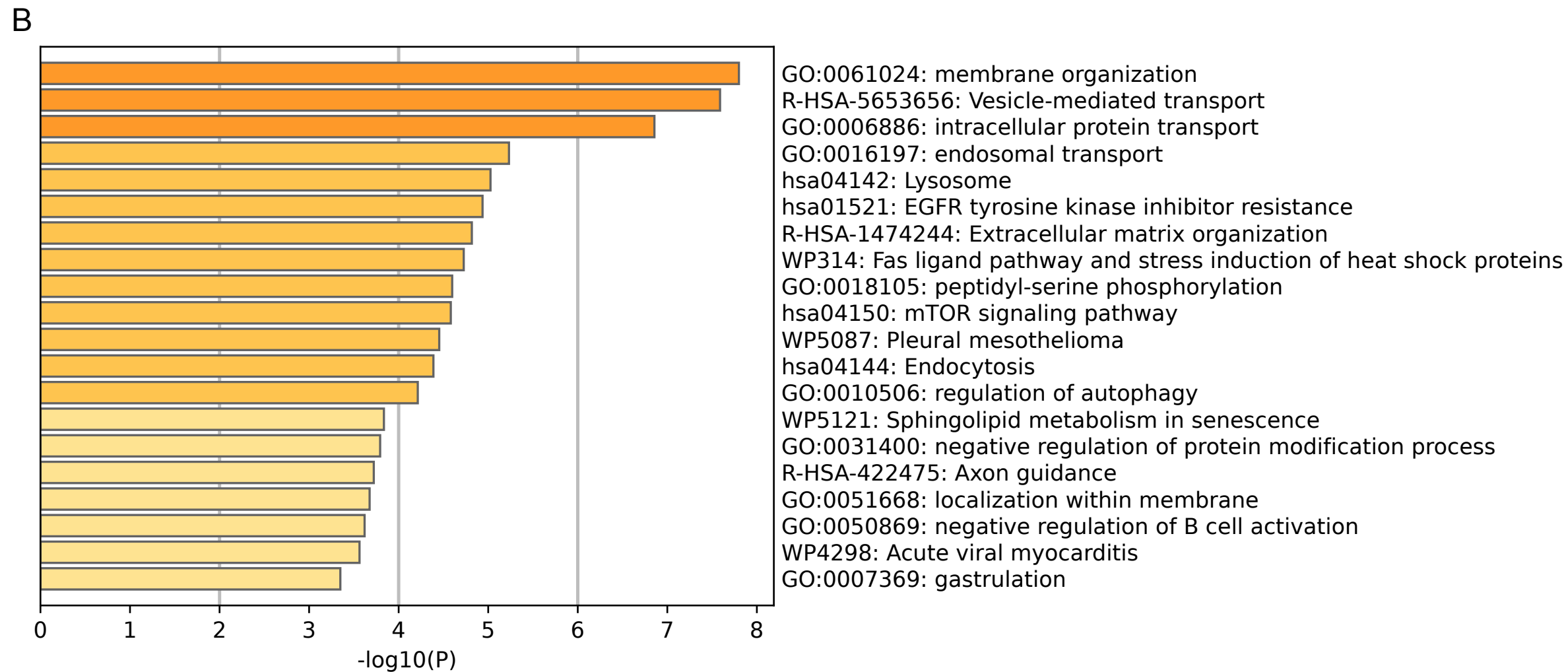

Supplement: Supplementary file 5 — Supporting information [file CTM2-15-e70160-s012.pdf]

A

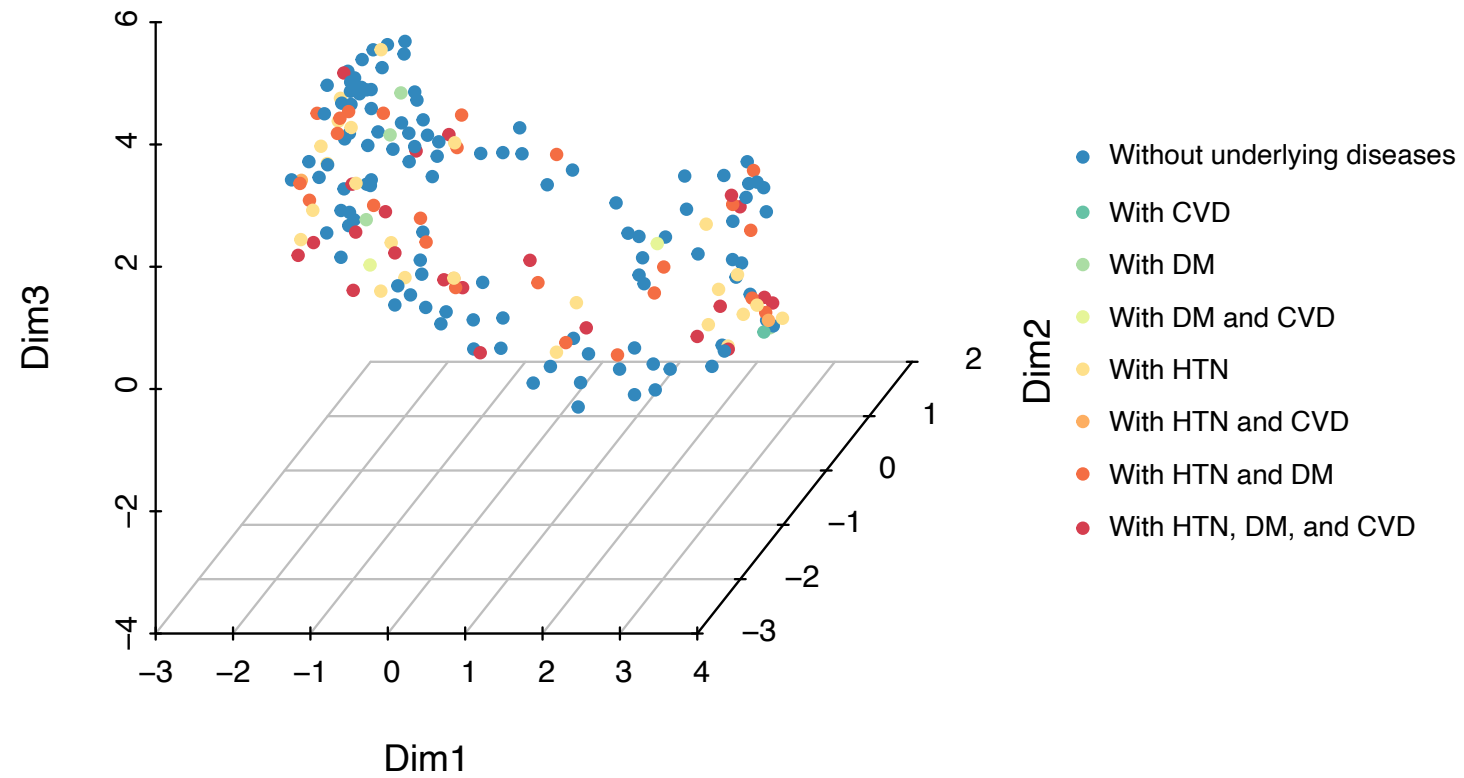

B

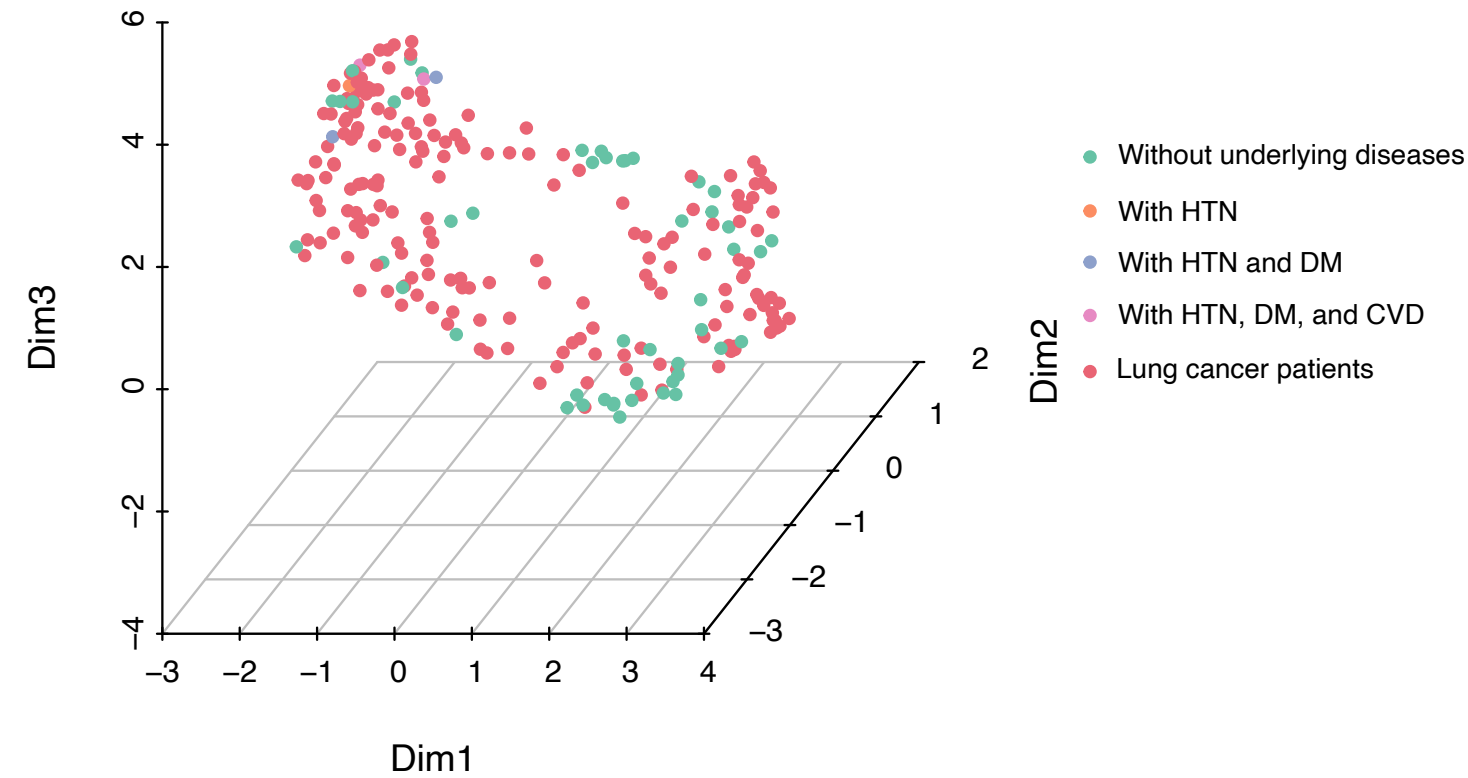

C

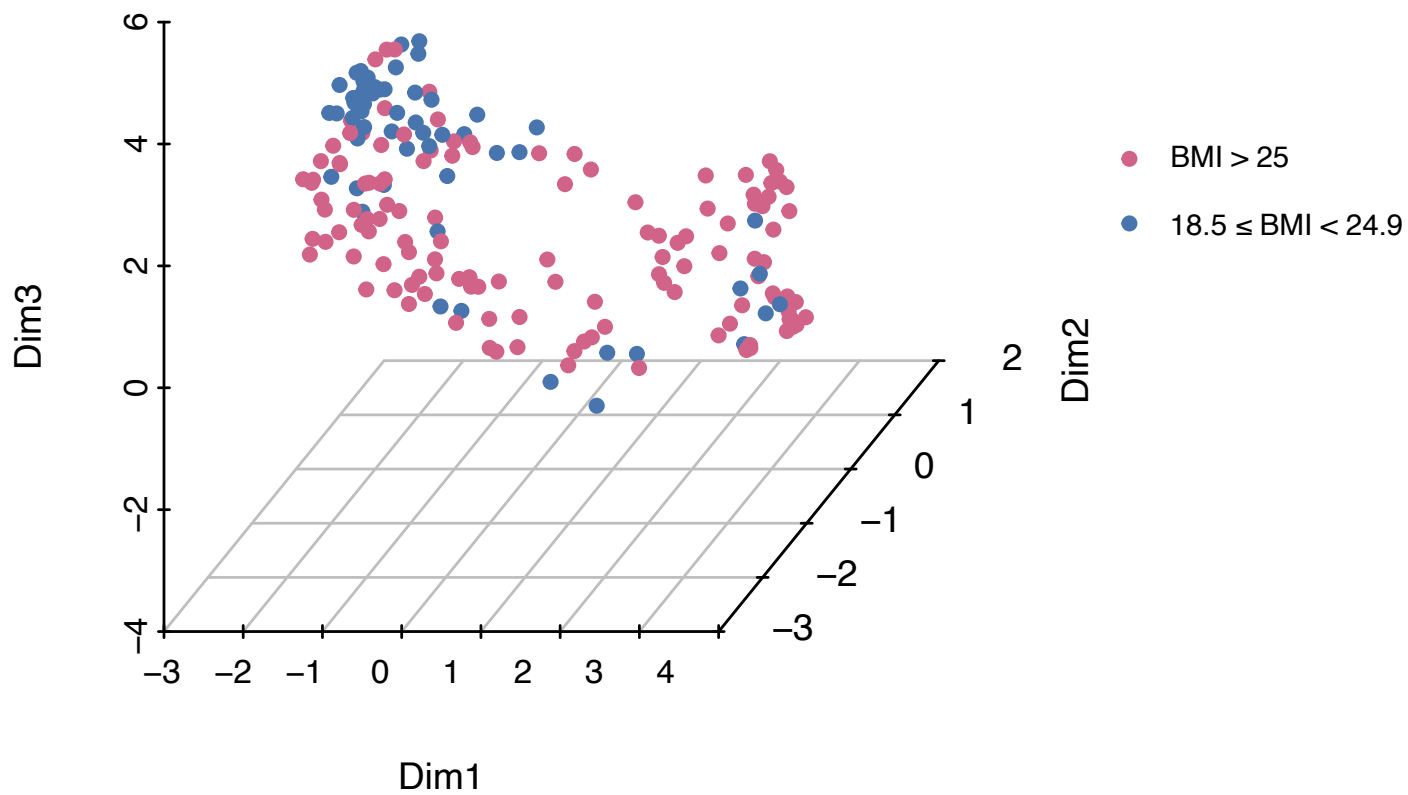

Supplement: Supplementary file 6 — Supporting information [file CTM2-15-e70160-s007.pdf]

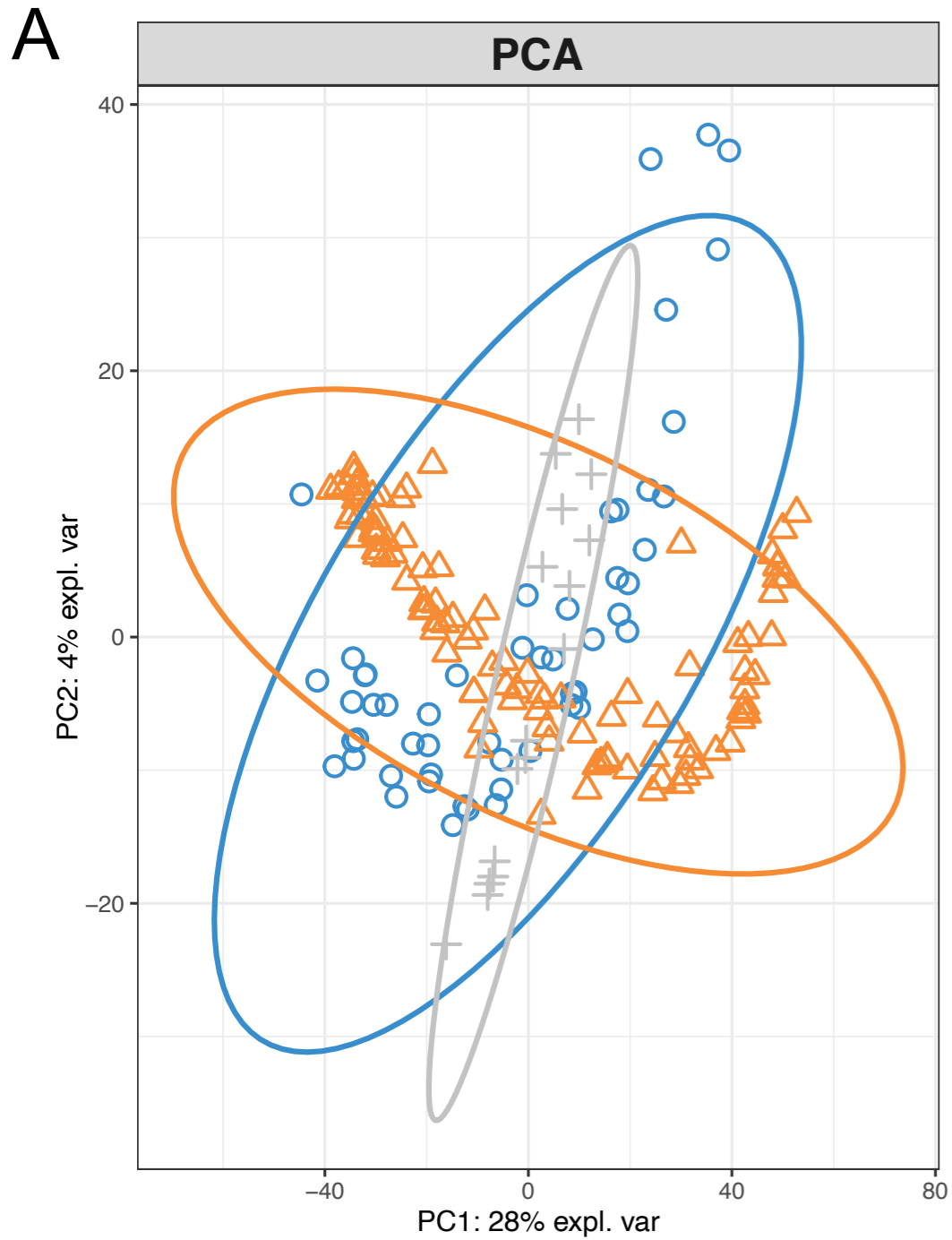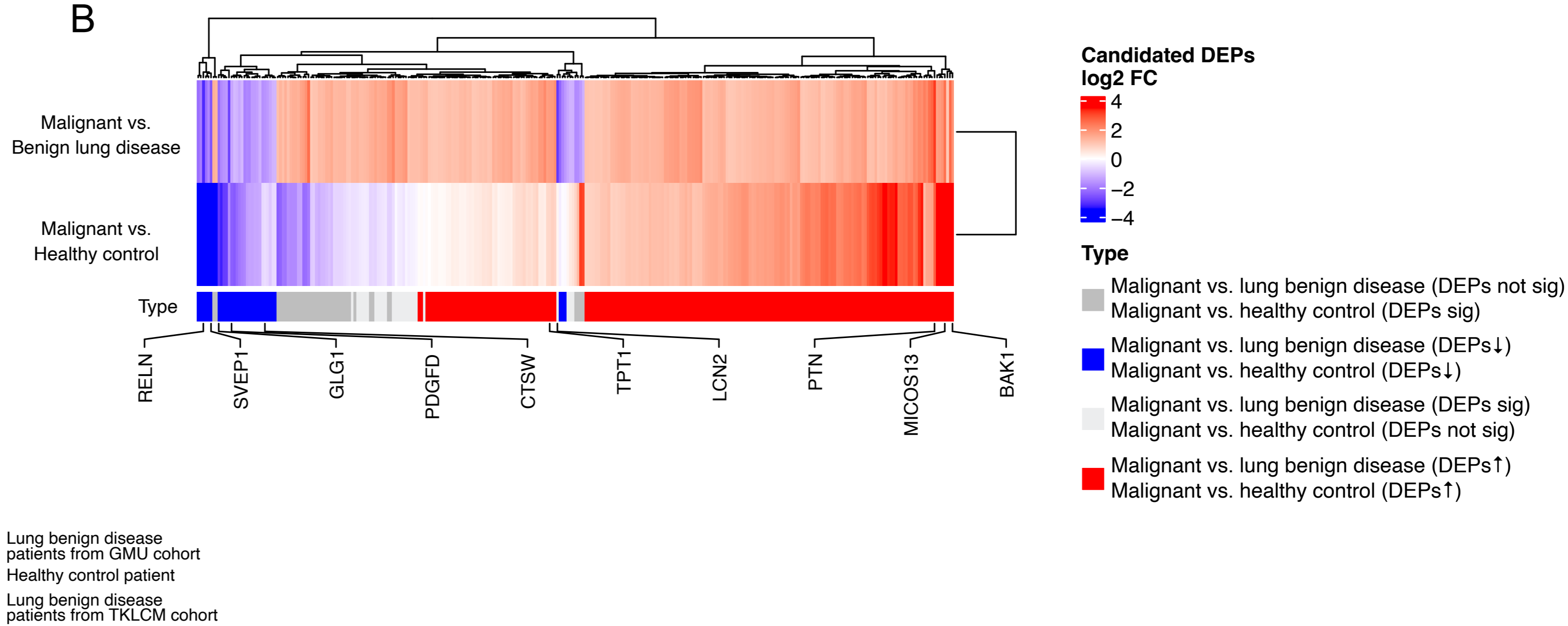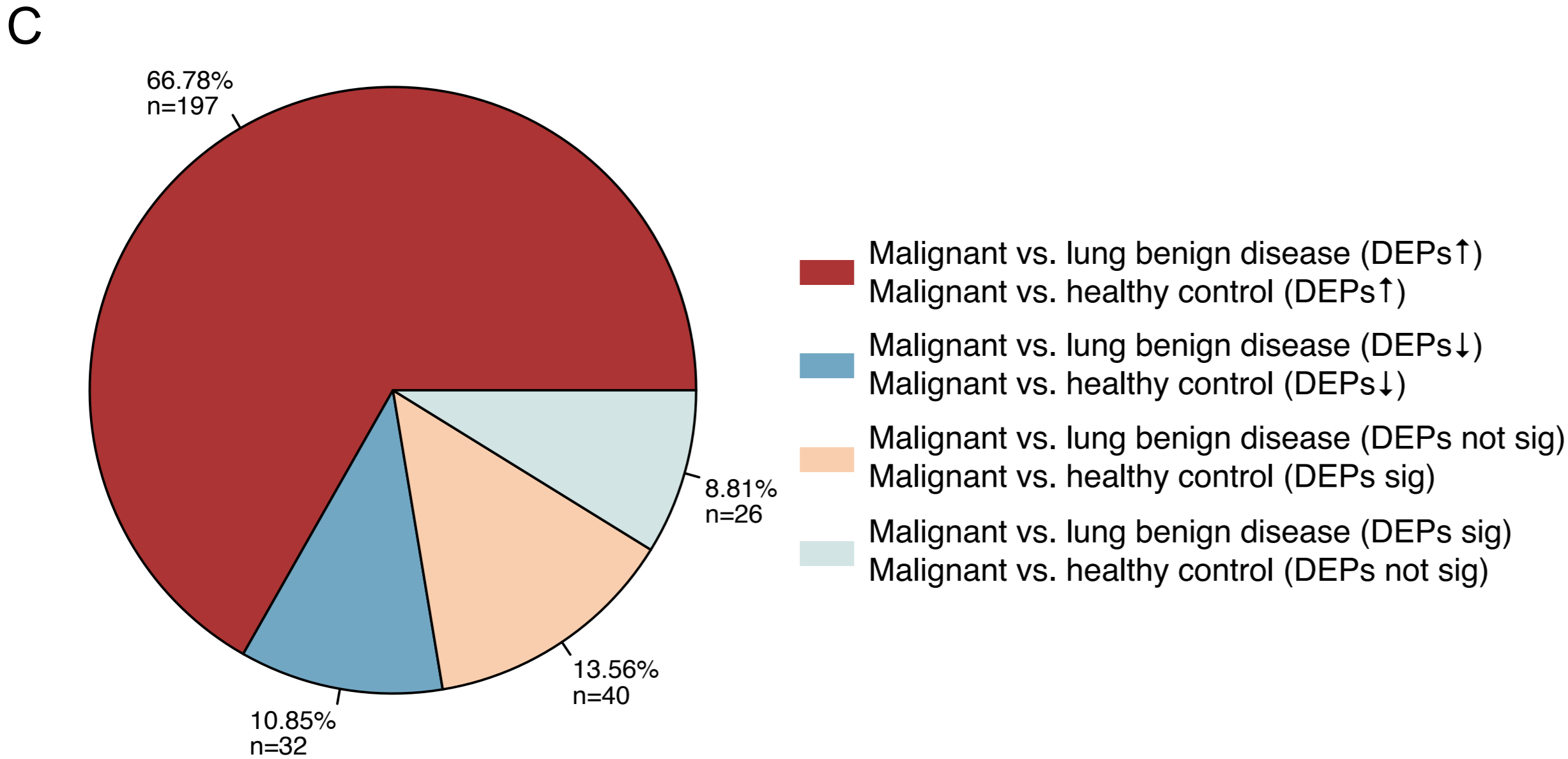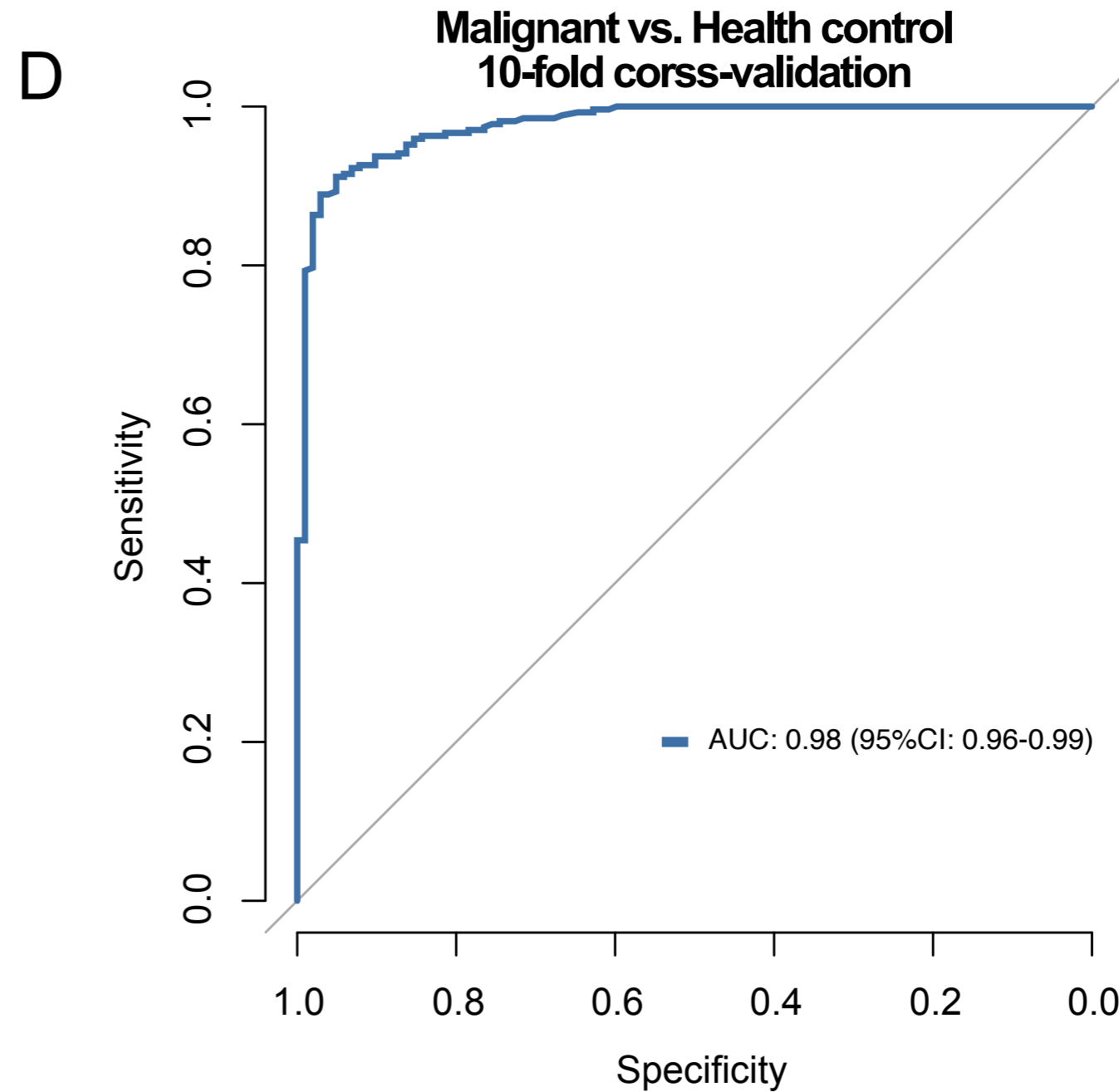

Supplement: Supplementary file 7 — Supporting information [file CTM2-15-e70160-s002.pdf]
