# Supplementary material for: LcProt: Proteomics‐based identification of plasma biomarkers for lung cancer multievent, a multicentre study
Source: Clin Transl Med. 2025 Jan 9;15(1):e70160. doi: 10.1002/ctm2.70160 (PMC11714244; doi:10.1002/ctm2.70160)
Supplement: Supplementary file 8 — Supporting information [file CTM2-15-e70160-s011.docx]

Supplementary Table 1. Baseline characteristic of participants in 1STGMU and NCRM cohort.

|  | 1STGMU cohort | | | | |  | NCRM cohort | | | | |
| --- | --- | --- | --- | --- | --- | --- | --- | --- | --- | --- | --- |
|  | Lung cancer patients  (n = 106) | |  | Lung benign diseases patients  (n = 21) | |  | Lung cancer patients  (n = 75) | |  | Lung benign diseases patients  (n = 26) | |
|  | Mean/N | SD/% |  | Mean/N | SD/% |  | Mean/N | SD/% |  | Mean/N | SD/% |
| Age (years) | 61.34 | 9.07 |  | 59.95 | 7.97 |  | 63.63 | 11.41 |  | 57.38 | 14.09 |
| Gender |  |  |  |  |  |  |  |  |  |  |  |
| Female | 39 | 36.79 |  | 5 | 23.81 |  | 26 | 34.67 |  | 5 | 19.23 |
| Male | 67 | 63.21 |  | 16 | 76.19 |  | 49 | 65.33 |  | 21 | 80.77 |
| Smoking status |  |  |  |  |  |  |  |  |  |  |  |
| Yes | 45 | 42.45 |  | 9 | 42.86 |  | 22 | 29.33 |  | 13 | 50.00 |
| No | 61 | 57.55 |  | 12 | 57.14 |  | 53 | 70.67 |  | 13 | 50.00 |
| Underlying diseases |  |  |  |  |  |  |  |  |  |  |  |
| Hypertension | 42 | 39.62 |  | 4 | 19.05 |  | 31 | 41.33 |  | 1 | 3.85 |
| Diabetes | 31 | 29.25 |  | 3 | 14.29 |  | 22 | 29.33 |  | 1 | 3.85 |
| CVD | 18 | 16.98 |  | 1 | 4.76 |  | 9 | 0.12 |  | 1 | 3.85 |
| Arrhythmia | 1 | 0.94 |  | 0 | 0 |  | 1 | 1.33 |  | 0 | 0 |
| COPD | 1 | 0.94 |  | 0 | 0 |  | 0 | 0 |  | 0 | 0 |
| Cancer | 0 | 0 |  | 0 | 0 |  | 1 | 1.33 |  | 0 | 0 |
| BMI (kg/m^2^) |  |  |  |  |  |  |  |  |  |  |  |
| 18.5 – 24.9 | 30 | 28.30 |  | - | - |  | 25 | 33.33 |  | - | - |
| > 25 | 76 | 71.70 |  | - | - |  | 50 | 66.67 |  | - | - |
| Tumor location |  |  |  |  |  |  |  |  |  |  |  |
| LUL | 27 | 25.47 |  | - | - |  | 21 | 28.00 |  | - | - |
| LLL | 14 | 13.21 |  | - | - |  | 11 | 14.67 |  | - | - |
| RUL | 34 | 32.08 |  | - | - |  | 19 | 25.33 |  | - | - |
| RML | 7 | 6.60 |  | - | - |  | 6 | 8.00 |  | - | - |
| RLL | 24 | 22.64 |  | - | - |  | 18 | 24.00 |  | - | - |
| T stage |  |  |  |  |  |  |  |  |  |  |  |
| 1b | 18 | 16.98 |  | - | - |  | 14 | 18.67 |  | - | - |
| 1c | 26 | 24.53 |  |  |  |  | 11 | 14.67 |  |  |  |
| 2a | 30 | 28.30 |  | - | - |  | 19 | 25.33 |  | - | - |
| 2b | 6 | 5.66 |  |  |  |  | 6 | 8.00 |  |  |  |
| 3 | 17 | 16.04 |  | - | - |  | 11 | 14.67 |  | - | - |
| 4 | 15 | 14.15 |  | - | - |  | 14 | 18.67 |  | - | - |
| N stage |  |  |  |  |  |  |  |  |  |  |  |
| 0 | 30 | 28.30 |  | - | - |  | 23 | 30.67 |  | - | - |
| 1 | 21 | 19.81 |  | - | - |  | 11 | 14.67 |  | - | - |
| 2 | 39 | 36.79 |  | - | - |  | 24 | 32.00 |  | - | - |
| 3 | 16 | 15.09 |  | - | - |  | 17 | 22.67 |  | - | - |
| M stage |  |  |  |  |  |  |  |  |  |  |  |
| 0 | 81 | 76.42 |  | - | - |  | 50 | 66.67 |  | - | - |
| 1a | 14 | 13.21 |  | - | - |  | 13 | 17.33 |  | - | - |
| 1b | 5 | 4.72 |  | - | - |  | 0 | 0.00 |  | - | - |
| 1c | 6 | 5.66 |  | - | - |  | 12 | 0.16 |  | - | - |
| Pathological stage |  |  |  |  |  |  |  |  |  |  |  |
| I | 22 | 20.75 |  | - | - |  | 19 | 25.33 |  | - | - |
| II | 30 | 20.30 |  | - | - |  | 15 | 20.00 |  | - | - |
| III | 29 | 27.36 |  | - | - |  | 16 | 21.33 |  | - | - |
| III | 25 | 23.58 |  | - | - |  | 25 | 33.33 |  | - | - |
| Genetic mutation |  |  |  |  |  |  |  |  |  |  |  |
| EGFR | 44 | 41.51 |  | - | - |  | 30 | 40.00 |  | - | - |
| ALK | 4 | 3.77 |  | - | - |  | 2 | 2.67 |  | - | - |
| KRAS | 6 | 5.66 |  | - | - |  | 4 | 5.33 |  | - |  |
| TP53 | 5 | 4.72 |  | - | - |  | 1 | 1.33 |  | - | -- |
| MET | 2 | 1.89 |  | - | - |  | 0 | 0.00 |  | - | - |
| ROS1 | 0 | 0.00 |  | - | - |  | 2 | 2.67 |  | - | - |
| Other rare mutation | 5 | 4.72 |  | - | - |  | 1 | 1.33 |  | - | - |
| None | 40 | 37.74 |  | - | - |  | 35 | 46.67 |  | - | - |
| Pathology |  |  |  |  |  |  |  |  |  |  |  |
| Adenocarcinoma | 73 | 68.87 |  | - | - |  | 62 | 82.67 |  | - | - |
| Squamous cell carcinoma | 18 | 16.98 |  | - | - |  | 9 | 12.00 |  | - | - |
| Small cell carcinoma | 3 | 2.83 |  | - | - |  | 3 | 4.00 |  | - | - |
| Other | 12 | 11.32 |  | - | - |  | 1 | 1.33 |  | - | - |

1STGMU: the First Affiliated Hospital of Guangzhou Medical University; NCRM: National Center for Respiratory Medicine; T: tumor; N: node; M: metastasis; LUL: Left Upper Lobe; LLL: Left Lower Lobe; RUL: Right Upper Lobe; RML: Right Middle Lobe; RLL: Right Lower Lobe; EGFR: Epidermal Growth Factor Receptor; ALK: Anaplastic Lymphoma Kinase; KRAS: Kirsten Rat Sarcoma Viral Oncogene Homolog; TP53: Tumor Protein 53; MET: Mesenchymal-Epithelial Transition Factor; ROS1: c-ros Oncogene 1.
