# Supplementary material for: LcProt: Proteomics‐based identification of plasma biomarkers for lung cancer multievent, a multicentre study
Source: Clin Transl Med. 2025 Jan 9;15(1):e70160. doi: 10.1002/ctm2.70160 (PMC11714244; doi:10.1002/ctm2.70160)
Supplement: Supplementary file 9 — Supporting information [file CTM2-15-e70160-s008.docx]

Supplementary Table 2. Baseline characteristic of health control participants.

|  | Mean/N | SD/% |
| --- | --- | --- |
| Gender |  |  |
| Female | 82 | 80.39 |
| Male | 20 | 19.61 |
| Ethnicity |  |  |
| Han | 99 | 97.06 |
| Other | 3 | 2.94 |
| Age | 54.14 | 7.64 |
| BMI | 25.21 | 2.61 |
| Blood pressure |  |  |
| Systolic blood pressure | 149.34 | 20.27 |
| Diastolic blood pressure | 88.10 | 19.49 |
| Smoking status |  |  |
| 1 | 9 | 8.82 |
| 2 | 93 | 91.18 |
| Region |  |  |
| East | 40 | 29.22 |
| Northwest | 39 | 38.24 |
| Northwest | 18 | 17.65 |
| Central | 3 | 2.94 |
| South | 2 | 1.96 |

BMI: body mass index.
