# Supplementary material for: LcProt: Proteomics‐based identification of plasma biomarkers for lung cancer multievent, a multicentre study
Source: Clin Transl Med. 2025 Jan 9;15(1):e70160. doi: 10.1002/ctm2.70160 (PMC11714244; doi:10.1002/ctm2.70160)
Supplement: Supplementary file 10 — Supporting information [file CTM2-15-e70160-s010.docx]

Supplementary Table 3. Detailed description of proteins in the final panels.

| Protein | Panel | Ensembl ID | UniProt | Description |
| --- | --- | --- | --- | --- |
| BAK1 | #1 | ENSG00000030110 | Q16611 | BCL2 antagonist/killer 1 |
| CTSW | #1 | ENSG00000172543 | P56202 | cathepsin W |
| MICOS13 | #1 | ENSG00000174917 | Q5XKP0 | mitochondrial contact site and cristae organizing system subunit 13 |
| RELN | #1 | ENSG00000189056 | P78509 | reelin |
| PTN | #1 | ENSG00000105894 | P21246 | pleiotrophin |
| TPT1 | #1 | ENSG00000133112 | P13693 | tumor protein, translationally-controlled 1 |
| SVEP1 | #1 | ENSG00000165124 | Q4LDE5 | sushi, von Willebrand factor type A, EGF and pentraxin domain containing 1 |
| PDGFD | #1 | ENSG00000170962 | Q9GZP0 | platelet derived growth factor D |
| LCN2 | #1 | ENSG00000148346 | P80188 | lipocalin 2 |
| GLG1 | #1 | ENSG00000090863 | Q92896 | golgi glycoprotein 1 |
| XPO1 | #2 | ENSG00000082898 | O14980 | exportin 1 |
| NPNT | #2 | ENSG00000168743 | Q6UXI9 | nephronectin |
| SND1 | #2 | ENSG00000197157 | Q7KZF4 | staphylococcal nuclease and tudor domain containing 1 |
| ARHGDIB | #2 | ENSG00000111348 | P52566 | Rho GDP dissociation inhibitor beta |
| RELCH | #2 | ENSG00000134444 | Q9P260 | RAB11 binding and LisH domain, coiled-coil and HEAT repeat containing |
| PDLIM1 | #2 | ENSG00000107438 | O00151 | PDZ and LIM domain 1 |
| PRKG1 | #2 | ENSG00000185532 | Q13976 | protein kinase cGMP-dependent 1 |
| MAPK14 | #2 | ENSG00000112062 | Q16539 | mitogen-activated protein kinase 14 |
| SPARC | #2 | ENSG00000113140 | P09486 | secreted protein acidic and cysteine rich |
| KPNB1 | #3 | ENSG00000108424 | Q14974 | karyopherin subunit beta 1 |
| COTL1 | #3 | ENSG00000103187 | Q14019 | coactosin like F-actin binding protein 1 |
| XPO1 | #3 | ENSG00000082898 | O14980 | exportin 1 |
| PTN | #3 | ENSG00000105894 | P21246 | pleiotrophin |
| TARBP1 | #3 | ENSG00000059588 | Q13395 | TAR (HIV-1) RNA binding protein 1 |
| DERL1 | #3 | ENSG00000136986 | Q9BUN8 | derlin 1 |
| CARD9 | #3 | ENSG00000187796 | Q9H257 | caspase recruitment domain family member 9 |
| SULF2 | #3 | ENSG00000196562 | Q8IWU5 | sulfatase 2 |
| PF4 | #3 | ENSG00000163737 | P02776 | platelet factor 4 |
| ARHGEF2 | #3 | ENSG00000116584 | Q92974 | Rho/Rac guanine nucleotide exchange factor 2 |
