# Supplementary material for: LcProt: Proteomics‐based identification of plasma biomarkers for lung cancer multievent, a multicentre study
Source: Clin Transl Med. 2025 Jan 9;15(1):e70160. doi: 10.1002/ctm2.70160 (PMC11714244; doi:10.1002/ctm2.70160)
Supplement: Supplementary file 11 — Supporting information [file CTM2-15-e70160-s004.docx]

The Guangzhou Medical University (GMU) cohort is a prospective cohort conducted by our center and supported by the China National Key Research and Development Program of China.

A total of nine center were participating in the cohort construction, the list of these centers was as follows: (1) the First Affiliated Hospital of Guangzhou Medical University; (2) National Center for Respiratory Medicine; (3) Cancer Hospital, Chinese Academy of Medical Sciences; (4) Chinese Academy of Medical Sciences & Peking Union Medical College; (5) Zhejiang Cancer Hospital; (6) West China Hospital, Sichuan University; (7) Zhongshan Hospital, Fudan University; (8) Second Affiliated Hospital of Zhejiang University School; (9) Tongji Hospital, Tongji Medical College, Huazhong University of Science and Technology.

This cohort included 2,757 participants with various lung diseases, comprising stage I–IV non-small cell lung cancer (NSCLC) patients, small cell lung cancer (SCLC) patients, patients with benign lung diseases, and healthy individuals undergoing routine physical examinations. NSCLC patients are stratified according to tumor stage and treatment protocol: early and intermediate-stage patients primarily undergo surgical treatment, locally advanced-stage and advanced-stage patients are primarily comprehensive treatment. Patients with limited-stage and extensive-stage SCLC to establish a comprehensive treatment cohort for SCLC were included. Additionally, patients with lung benign disease were also included in this cohort.

Peripheral blood samples, tumor tissues, paired para-cancerous tissues, and biopsy tissue samples are collected before and after treatment, based on the treatment protocol and disease stage. For patients eligible for surgery, samples are collected both preoperatively and postoperatively. For patients undergoing comprehensive treatment, peripheral blood and biopsy tissue samples are collected before and after treatment. Additionally, peripheral blood samples are collected from patients with benign lung diseases and from healthy individuals undergoing routine physical examinations at each participating center.
