# Supplementary material for: LcProt: Proteomics‐based identification of plasma biomarkers for lung cancer multievent, a multicentre study
Source: Clin Transl Med. 2025 Jan 9;15(1):e70160. doi: 10.1002/ctm2.70160 (PMC11714244; doi:10.1002/ctm2.70160)
Supplement: Supplementary file 12 — Supporting information [file CTM2-15-e70160-s006.docx]

**Standard Operating Procedure for Plasma Separation**

**1. Purpose**

The purpose of this Standard Operating Procedure (SOP) is to standardize the procedure for plasma separation from blood samples collected.

**2. Scope**

This SOP applies to the separation of plasma specimens from whole blood collected with anticoagulants.

**3. Definitions**

3.1 Plasma Definition

Plasma refers to the liquid component of whole blood that is obtained after anticoagulation and centrifugation. It contains fibrinogen and is free of cellular elements.

3.2 Responsibilities

Laboratory Technicians are responsible for executing the procedure as described.

The Laboratory Supervisor is responsible for overseeing the implementation of this SOP.

**4. Equipment and Materials**

4.1 Personal Protective Equipment (PPE)

Gloves, masks, laboratory protective clothing, goggles, and other relevant protective equipment.

Waste container with bleach solution

4.2 Containers

Sterile and nuclease-free cryovials

4.3 Instruments and Equipment

Beckman Coulter Allegra X-15R Centrifuge (B00340AA)

Eppendorf Research Plus 1ml pipette with corresponding 1ml sterile and nuclease-free pipette tips

Refrigerator or cooling box (4°C)

Ultra-low temperature freezer (-80°C)

**5. Procedure**

5.1 Sample Collection

Perform a venipuncture on the antecubital vein of the patient in a fasting state.

Collect 10ml of venous blood in an EDTA blood collection tube.

5.2 Centrifugation

The collected blood sample should be processed within 4 hours.

Centrifuge at 1600g, 4°C, for 15 minutes.

5.3 Plasma Aliquoting

The upper clear layer represents the plasma needed for further processing after centrifugation.

Carefully extract the plasma using a pipette and aliquot it into sterile 2ml cryovials.

5.4 Storage

Immediately place the aliquoted cryovials in a -80°C freezer for storage until needed.

**6. Safety Considerations:**

Laboratory Technicians must wear personal protective equipment at all times during the procedure.

Blood samples should be centrifuged as soon as possible. If immediate centrifugation is not feasible, store the samples under low-temperature conditions (in a 4°C refrigerator) for no more than 4 hours.

Ensure safety when using the centrifuge by maintaining its balance.

Minimize exposure of samples to the air during processing to reduce the risk of contamination.

Pipette tips and cryovials must be sterile and nuclease-free to prevent contamination.
