# Supplementary material for: LcProt: Proteomics‐based identification of plasma biomarkers for lung cancer multievent, a multicentre study
Source: Clin Transl Med. 2025 Jan 9;15(1):e70160. doi: 10.1002/ctm2.70160 (PMC11714244; doi:10.1002/ctm2.70160)
Supplement: Supplementary file 13 — Supporting information [file CTM2-15-e70160-s014.docx]

**The nano-low abundant plasma enrichment/mass spectrometry ^TM^ (n-LAPE/MS^TM^) preparation**

Zeolite bead NaY was synthesized using the n-LAPE/MS^TM^ (nano-low abundant plasma enrichment/mass spectrometry ^TM^) kit through gel formulation and calcination. Each 40 µL plasma sample was co-incubated with 1 mg of pre-mixed beads for 30 minutes at room temperature, followed by centrifugation at 12,000 g for 5 minutes. The supernatant was discarded, and the enriched beads were washed three times with 500 µL of Tris-EDTA ((10 mM Tris-HCl (tris(hydroxymethyl)aminomethane-hydrochloride), 1 mM EDTA (Ethylenediaminetetraacetic acid)).

The beads were then resuspended in 50 µL of lysis buffer containing TCEP (tris (2-

carboxyethyl) phosphine hydrochloride) and CAA(2-Chloroacetamide), heated at 95 °C for 10 minutes with shaking. After cooling to room temperature, 2 µL of trypsin digestion buffer was added, and the mixture was incubated at 37 °C for 4 hours with shaking. The peptides were precipitated with 950 µL of ACN (acetonitrile) and desalted using the SP3 method (Single-Pot Solid-Phase-Enhanced Sample Preparation method) on beads. The peptides were eluted with 20 µL of elution buffer. Finally, the peptide eluate was dried and prepared for subsequent LC-MS/MS (liquid chromatography-tandem mass spectrometry) detection.
