# Supplementary material for: LcProt: Proteomics‐based identification of plasma biomarkers for lung cancer multievent, a multicentre study
Source: Clin Transl Med. 2025 Jan 9;15(1):e70160. doi: 10.1002/ctm2.70160 (PMC11714244; doi:10.1002/ctm2.70160)
Supplement: Supplementary file 14 — Supporting information [file CTM2-15-e70160-s001.docx]

This work involves multiple cohorts, the evaluation results of the QC-SP and the QC-MS for both cohorts are as follows:

**QC-SP**

The work involves two cohorts: the development cohort and the validation cohort. The development cohort includes 241 samples, including a total of 12 sample preparation quality controls (23 samples + 1 control), named: QC-SP-DC-x (quality control-sample preparation-development cohort-number). The validation cohort includes 46 samples, with a total of 2 sample preparation quality controls, named: QC-SP-VC-x (quality control-sample preparation-validation cohort-number).


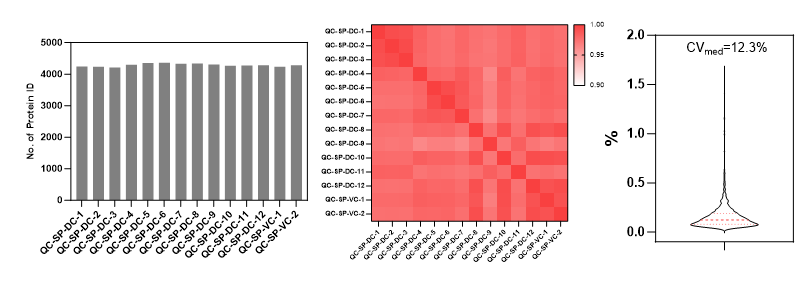


As shown in the figure above, the 14 QC-SP samples involved in the sample preparation process of 287 samples exhibited stable protein ID identification numbers with no significant differences. Meanwhile the protein quantification correlation was above 0.96, the median coefficient of variation (CV) of the protein quantification values from the 14 QC-SP samples was only 12.3%. These further indicate that the sample preparation process is stable, with no significant batch-to-batch variations.

**QC-MS**

The MS instrument quality control also involves two cohorts. The development cohort includes 241 samples, with a total of 13 instrument quality controls (20 samples + 1 control), named: QC-MS-DC-x (quality control-mass spectrometry-development cohort-number). The validation cohort includes 46 samples, with a total of 3 instrument quality controls, named: QC-MS-VC-x (quality control-mass spectrometry-validation cohort-number).


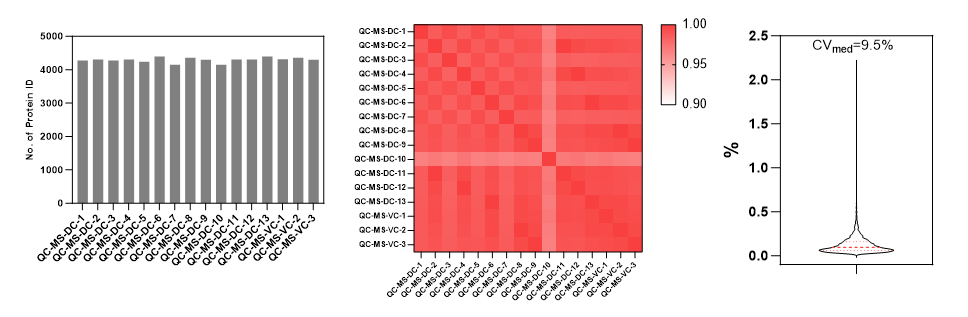


As shown in the figure above, the 16 QC-MS samples involved in the MS detection process of 287 samples exhibited stable protein ID identification numbers with no significant differences. Meanwhile the protein quantification correlation was above 0.96, and the median coefficient of variation (CV) of the protein quantification values from the 16 QC-MS samples was only 9.5%. These further indicate that the sample detection process is stable, with no significant batch-to-batch variations.
